# Thermodynamics and Kinetics in Host Design for Dendrite‐Free Zinc Metal Anodes

**DOI:** 10.1002/advs.77270

**Published:** 2026-08-21

**Authors:** Peng Xiao Sun, Quanwei Ma, Yangyang Liu, Longhai Zhang, Rui Wang, Yu‐Hui Zhu, Tengfei Zhou, Sen Xin, Le Yu, Chaofeng Zhang

**Affiliations:** ^1^ School of Materials Science and Engineering Institutes of Physical Science and Information Technology Leibniz International Joint Research Centre of Materials Sciences of Anhui Province Anhui University Hefei People's Republic of China; ^2^ Laboratory of Molecular Nanostructure and Nanotechnology CAS Research/Education Center for Excellence in Molecular Sciences Beijing National Laboratory for Molecular Sciences Institute of Chemistry Chinese Academy of Sciences (CAS) Beijing People's Republic of China; ^3^ School of Chemical Sciences University of Chinese Academy of Sciences Beijing People's Republic of China; ^4^ State Key Lab of Organic‐Inorganic Composites Beijing University of Chemical Technology Beijing People's Republic of China

**Keywords:** aqueous battery, host design, reaction kinetics, reaction thermodynamics, Zn metal anode

## Abstract

Aqueous zinc metal batteries (AZMBs) have emerged as promising candidates for grid‐scale energy storage due to their intrinsic safety, cost‐effectiveness, and environmental friendliness. Nonetheless, the practical application of AZMBs is restricted by the uncontrolled Zn dendrite growth arising from undesirable reaction kinetics, as well as severe parasitic reactions (e.g., hydrogen evolution reaction, corrosion, and passivation) caused by the thermodynamic instability of Zn metal anodes (ZMAs) in aqueous electrolytes. Among various strategies, host design enables the effective mitigation of the aforementioned challenges through modulating thermodynamic properties (e.g., surface energy, adsorption energy, and nucleation energy barrier) and kinetic parameters (e.g., diffusion and desolvation of hydrated Zn^2+^ ions, and charge transfer). This review comprehensively summarizes the innovative advances in host design strategies and especially elucidates their underlying design principles from the insights of thermodynamics and kinetics. To be specific, the host design strategies are systematically categorized into surface modification, interface engineering, and spatial confinement. Finally, perspectives and recommendations for the further development of advanced hosts toward ultra‐stable ZMAs are proposed.

## Graphical abstract

This review comprehensively summarizes recent advances in host design strategies for high‐performance zinc metal anodes, and deeply elucidates their thermodynamic and kinetic design principles from the perspective of surface modification, interface engineering, and spatial confinement. Based on in‐depth insights into thermodynamics and kinetics, perspectives and recommendations are presented to further develop advanced hosts for ultra‐stable zinc metal anodes.

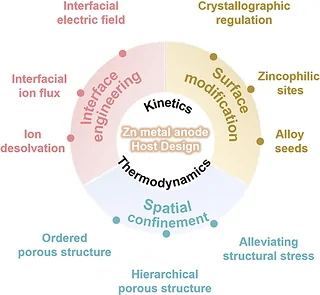

## 1 Introduction

The ever‐growing energy demands and the depletion of fossil‐fuel resources have stimulated burgeoning enthusiasm of researchers toward the integration of renewable energy [1, 2, 3, 4, 5, 6]. Among all of the electrochemical energy storage systems (EESs), the lithium (Li) ion battery has been generationally developed, achieving a dominant role in the energy‐storage technologies currently [7, 8, 9, 10, 11, 12, 13, 14, 15, 16, 17, 18, 19, 20]. However, the great obstructions toward the ever‐increasing cost of Li resources, low theoretical specific capacity of graphite anodes, and security problems have spurred extensive research on affordable energy harvesting and storage technology [21, 22, 23, 24]. Currently, aqueous zinc (Zn) metal batteries (AZMBs) are expected to be one of the desirable candidates for next‐generation EESs on account of their superiorities of low cost, environmental friendliness, and intrinsic safety [25, 26, 27, 28, 29, 30, 31, 32, 33, 34].

**FIGURE 1:**
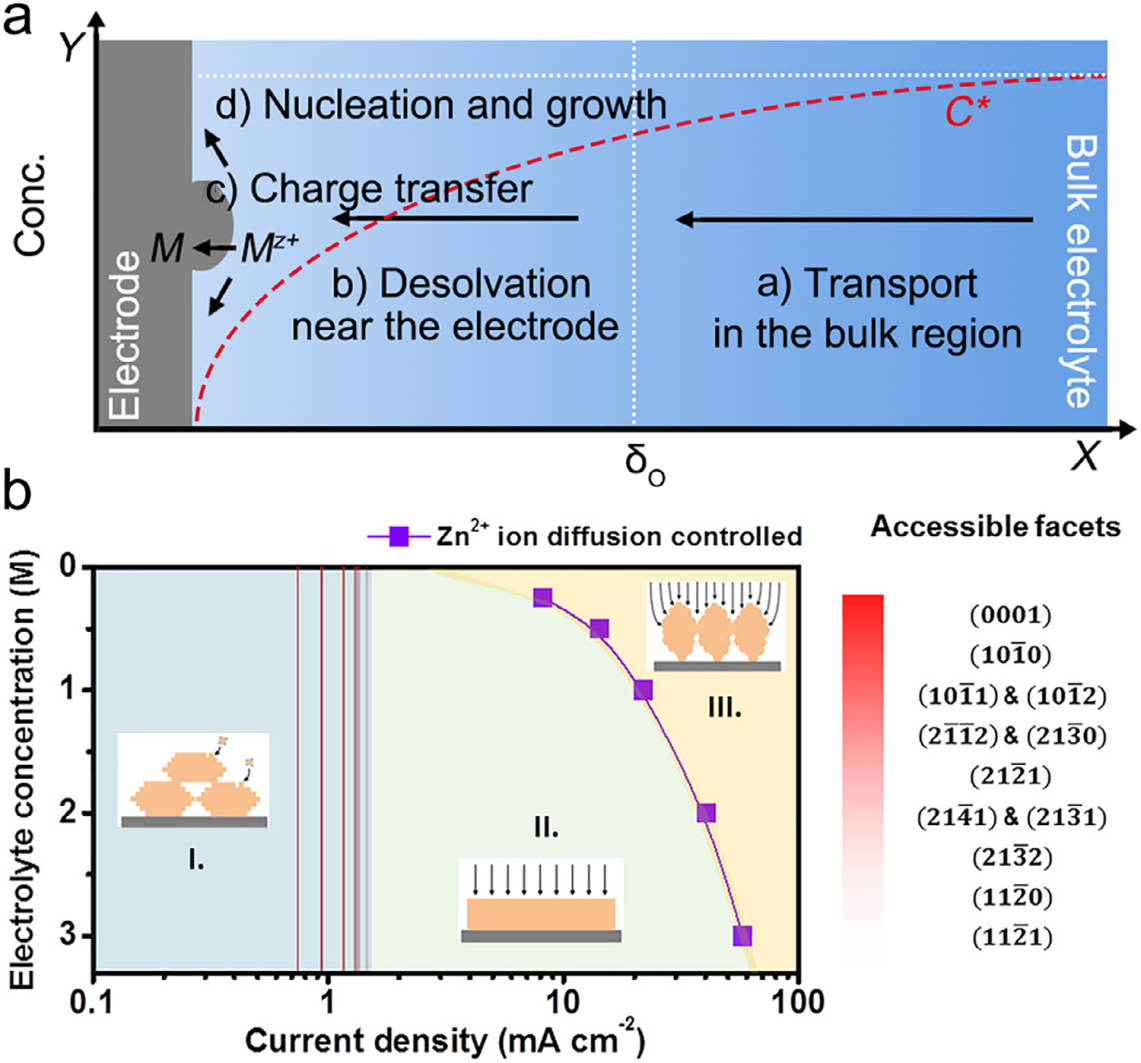
(a) Schematic diagram of Zn deposition process [43]. Copyright 2021, American Association for the Advancement of Science. (b) Illustration of different morphology evolution modes of Zn deposits with different conditions [51]. Copyright 2022, Wiley‐VCH.

Metallic Zn is widely recognized as an ideal anode for AZMBs owing to its abundant reserves, high theoretical capacity (820 mA h g^−1^ and 5855 mAh cm^−3^), and low redox potential (−0.76 V vs. SHE). Nevertheless, uncontrollable Zn dendrite growth and detrimental parasitic reactions (e.g., the hydrogen evolution reaction (HER), electrochemical corrosion, and passivation) have hampered the widespread application of AZMBs [3, 35, 36, 37, 38, 39, 40]. These inherent drawbacks of Zn metal anodes (ZMAs) mainly stem from the thermodynamic instability of metallic Zn in aqueous electrolytes and inferior kinetic behavior during Zn deposition [41, 42]. Generally, the whole Zn deposition process can be divided into four steps: hydrated Zn^2+^ ions migration in the electrolyte and desolvation at the electrode/electrolyte interface, Zn^2+^ ions adsorption on the electrode surface and charge transfer, Zn nucleation at energetically favorable sites, and subsequent growth (Figure 1a) [43]. Zn dendrite growth is primarily attributed to unfavorable thermodynamic properties and undesirable interfacial reaction kinetics [44, 45, 46]. As for commercial Zn foil, the defects and cracks featuring high surface energy and strong adsorption for Zn^2+^ generally induce a preferential Zn nucleation due to their low nucleation energy barrier (Δ*G*_nucleation_) [45]. Moreover, the deposition reaction rate of these Zn nuclei is significantly accelerated due to the intensified local electric field and accumulated Zn^2+^ concentration, thereby inevitably inducing uneven Zn deposition and subsequent dendrite growth [38, 47]. The accumulation of structural stress during Zn deposition also leads to the generation of microcracks and drives Zn dendrite growth, ultimately causing severe structural collapse [48, 49]. Moreover, the migration and desolvation of hydrated Zn^2+^ ions have been considered as the rate‐determining step of the deposition reaction [36, 50]. The sluggish diffusion and desolvation of hydrated Zn^2+^ ions induce a distinct concentration polarization under high current density, which triggers preferential deposition on dendrite tips and accelerates dendritic growth (Figure 1b) [51, 52]. Ultimately, rampant growth of sharp dendrites pierces separator and gives rise to an internal short circuit [53, 54]. Meanwhile, preferential dissolution at the root of Zn dendrites leads to detachment of active Zn from the electrode surface, thus forming “dead Zn” and causing serious capacity attenuation [55, 56, 57, 58]. The inherent thermodynamic instability of the ZMA‐electrolyte interface triggers undesirable parasitic reactions including HER, corrosion, and passivation [41, 59, 60, 61]. Furthermore, desolvation of hydrated Zn^2+^ boosts the H_2_O splitting at the ZMA‐electrolyte interface and further aggravates HER [36]. Meanwhile, H_2_ evolution increases the OH^−^ ion concentration at the ZMA‐electrolyte interface, enabling the formation of inert passivation layers (e.g., ZnO, Zn(OH)_2_, and Zn_4_SO_4_(OH)_6_·*x*H_2_O) on the ZMA surface [62]. H_2_ evolution and the formation of passivation layers inevitably consume active Zn, causing a rapid capacity attenuation and inner pressure surge [63, 64, 65]. Moreover, they cause uneven electric field distribution, sluggish ion diffusion, and increased charge transfer resistance, deteriorating interfacial reaction kinetics and accelerating the growth of Zn dendrites [66, 67, 68, 69, 70].

**FIGURE 2:**
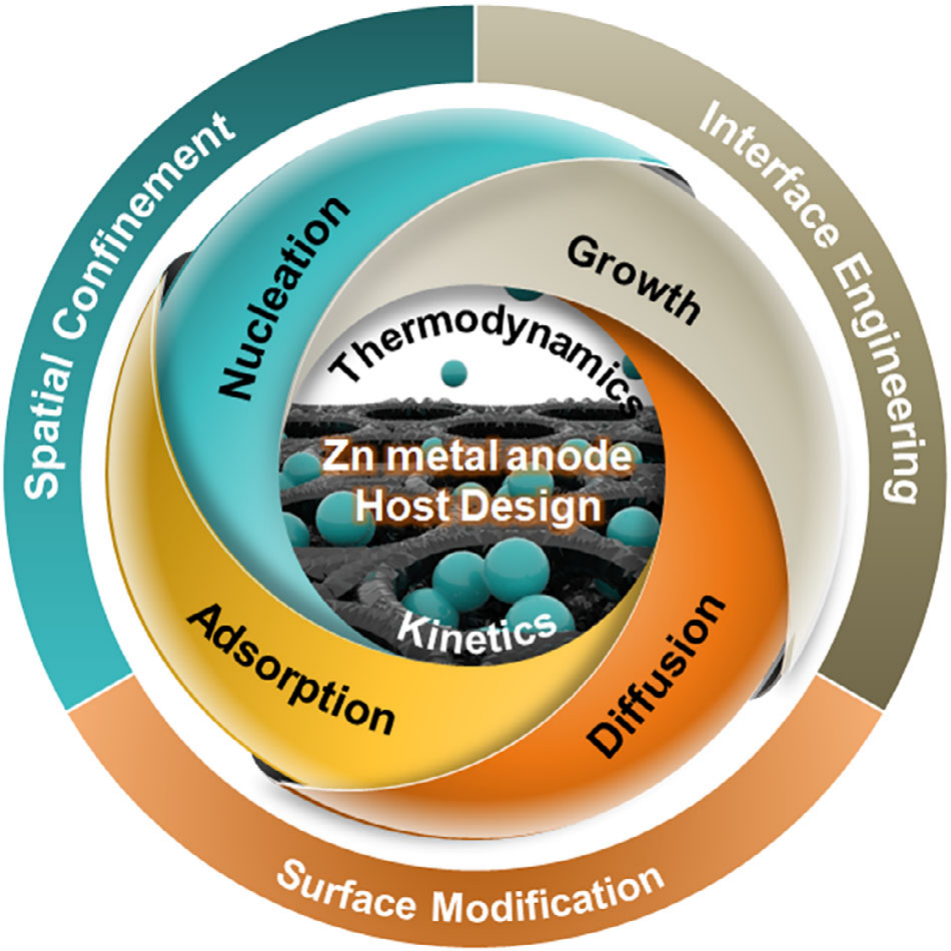
Synergism of thermodynamic and kinetic manipulation in host designs for high‐performance ZMAs.

Until now, numerous rational strategies have been proposed to suppress the Zn dendrites and parasitic reactions, which can be categorized into artificial interphase layer construction, electrolyte optimization, and host design [43, 71, 72, 73]. Among these strategies, host design can synergistically enhance the thermodynamic stability of ZMAs and optimize deposition kinetics by surface modification, interfacial engineering, and spatial confinement (Figure 2) [66, 74, 75, 76, 77, 78, 79]. From the perspective of surface modification, anchoring plentiful zincophilic species on host materials with strong adsorption energy for Zn^2+^ ions can effectively reduce the Δ*G*_nucleation_ [77, 80]. The zincophilic species not only provide sufficient nucleation sites for homogeneous Zn nucleation, but also lower Zn nucleation overpotential to achieve effective inhibition of HER [77, 81, 82, 83]. Additionally, several zincophilic species can increase the overpotential of HER and improve the thermodynamic stability of ZMAs [76, 84, 85, 86, 87, 88, 89, 90]. Moreover, Zn(002) crystal plane features a low surface energy, preferential lateral growth tendency, and superior chemical stability, which facilitates the formation of thermodynamically stable deposition layers [88]. The oriented growth of Zn (002) crystal planes can be achieved by constructing a host surface with high lattice matching, which is recognized as a pivotal approach for dendrite‐free and long‐lifespan ZMAs [85, 91, 92, 93, 94]. Nevertheless, surface modification strategies fail to provide effective protection for subsequently grown Zn deposits exposed to the electrolyte [95, 96]. Interfacial microenvironment reconstruction utilizing interface engineering can induce uniform Zn deposition. In particular, structural regulation of a 3D zincophilic host can homogenize the interfacial electric field, lower local current density, and optimize interfacial ion flux for reconstructing the electrode microenvironment. Furthermore, constructing functional interfacial layers on the host surface can redistribute the interfacial electric field, modulate local ion concentration, and accelerate the desolvation of hydrated Zn^2+^ ions, thereby optimizing interfacial reaction kinetics and enabling homogeneous Zn deposition [51, 95, 97, 98]. Furthermore, functional interfacial layers remarkably suppress water‐induced parasitic reactions by shielding the electrolyte, greatly boosting the thermodynamic stability of ZMAs [3, 45, 48, 99, 100, 101, 102]. However, drastic volume variations and structural stress induced by Zn plating/stripping can break the functional interfacial layer [15, 103]. 3D hollow hosts with tunable pore sizes and ordered structures can optimize electric field distribution and lower local current density, thus fully utilizing surface zincophilic sites and achieving precise physical confinement [75, 81, 104, 105]. Furthermore, structure stress arising from the volume expansion during the Zn plating/stripping process is alleviated by the sufficient space within the host [2, 15, 23]. By constructing hierarchical structures with gradient‐distributed compositions and pore sizes, desired 3D hosts can effectively induce Zn deposition within the frameworks, thereby sufficiently relieving dendrite growth and parasitic reactions [78, 80]. Although increasing efforts have been made to address the above obstacles, the host design for ZMA is still challenging and requires comprehensive insights into thermodynamics and kinetics as a guideline.

In this review, we thoroughly clarify the fundamental thermodynamic and kinetic principles of Zn deposition, and focus on analyzing dominant thermodynamic factors (e.g., Δ*G*_nucleation_, surface energy, and adsorption energy) and pivotal kinetic factors (e.g., migration and desolvation of hydrated Zn^2+^ ions, and interfacial charge transfer). Then, we comprehensively summarize the innovative advances in host design strategies for high‐performance ZMAs, and further elaborate the thermodynamic and kinetic design principles applicable to surface modification, interface engineering, and spatial confinement. Finally, perspectives and suggestions for advanced host designs are presented to guide future research toward ultra‐stable ZMAs with both superior sustainability and capacity.

## 2 The Thermodynamic and Kinetic Challenges of ZMAs

### 2.1 The Thermodynamic Obstacles for ZMAs

**FIGURE 3:**
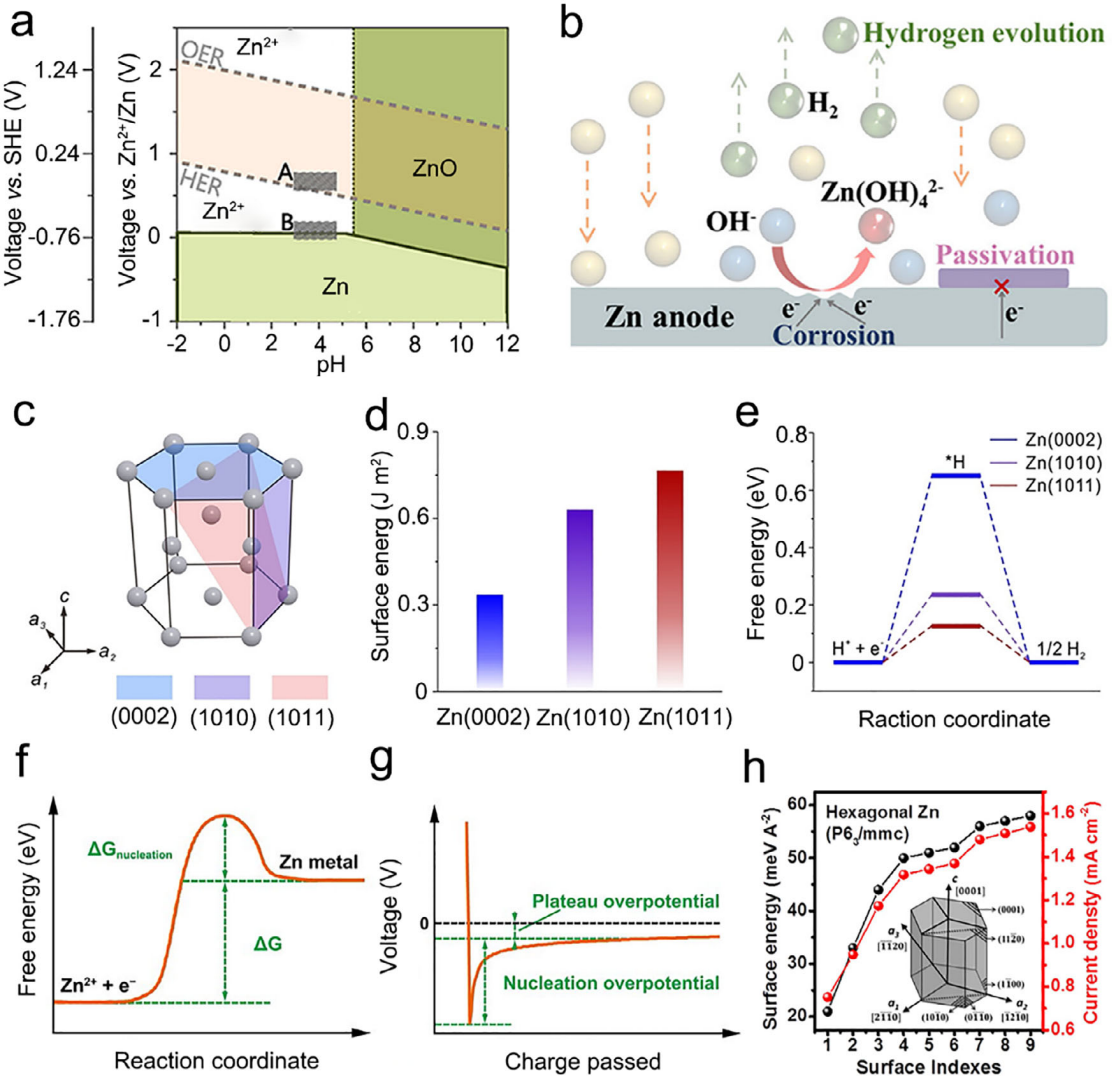
(a) Pourbaix diagram of HER and oxygen evolution reaction in aqueous solutions [106, 111]. Copyright 2021, American Chemical Society. (b) Schematic illustration of undesired reactions of ZMAs [110]. Copyright 2021, American Chemical Society. (c) Schematic diagram of the crystallographic orientations of various planes. (d) The surface energies and (e) HER energy barriers of the various planes of Zn [86]. Copyright 2025, Wiley‐VCH. (f) The schematic diagram of the relationship between 𝛥*G*_nucleation_ and free energy. (g) The typical voltage profiles of overpotential on galvanostatic Zn deposition [123]. Copyright 2020, Wiley‐VCH. (h) Surface energy and electrochemical activation current density of various crystal planes of metallic Zn [51]. Copyright 2022, Wiley‐VCH.

Substantial research efforts have been devoted to addressing the arguably more fundamental thermodynamic principle of ZMAs and their impacts on the nucleation and growth of Zn deposits during battery charge/discharge processes, which are of pivotal significance for the design of advanced AZMBs [3, 44, 50]. According to the Pourbaix diagram, the Zn^2+^/Zn equilibrium potential is invariably lower than that of H_2_O/H_2_ across the entire pH spectrum, indicating a thermodynamic predisposition of HER to precede Zn deposition (Figure 3a) [60, 106, 107, 108]. Meanwhile, the continuous consumption of H^+^ ions by the HER elevates the local pH at the electrode/electrolyte interface due to the accumulation of OH^−^ ions [109]. This alkaline shift subsequently drives the preferential precipitation of irreversible by‐products (e.g., Zn_4_SO_4_(OH)_6_·*x*H_2_O, Zn(OH)_2_ and ZnO), which actively form passivation layers on the electrode surface (Figure 3b) [30, 110]. Another manifestation of the intrinsic thermodynamic instability of metallic Zn is the susceptibility to corrosion in aqueous electrolytes via direct chemical attack by water molecules, leading to the formation of Zn(OH)_2_ and H_2_ gas [23, 44]. Notably, interfacial corrosion and the HER are exacerbated when ZMAs operate at more negative potentials during Zn deposition [111]. The above parasitic reactions continuously deplete active materials and severely compromise Coulombic efficiency (CE) and cycling stability of ZMAs. Therefore, a pivotal bottleneck must be addressed for the successful commercialization of AZMBs [56, 57].

The thermodynamic properties of ZMAs are also governed by their crystallographic characteristics, which fundamentally govern the stability and reversibility of ZMAs. A direct correspondence exists between the crystallographic texture and grain orientation. Zn(0002), Zn(1010), and Zn(1011) textured growth can cause initial Zn grains to align parallel, perpendicular, and obliquely to the conductive substrate, respectively (Figure 3c) [86]. Among the above crystal faces, the Zn(0002) crystal face featuring the high atomic planar packing density exhibits the lowest surface energy, contributing to forming the thermodynamically stable Zn deposits (Figure 3d) [112, 113, 114, 115]. Additionally, the low self‐diffusion energy barrier of Zn atoms on the Zn(0002) crystal face thermodynamically favors lateral diffusion, enabling the formation of an atomically smooth and highly oriented Zn crystalline layer on the electrode surface [3, 116]. Moreover, the scarcity of unsaturated bonds within the Zn(0002) configuration endows the ZMAs with a low adsorption energy for H^*^ and elevates the energy barrier for HER, thereby imparting enhanced resistance against parasitic side reactions (Figure 3e) [85, 91]. Conversely, Zn deposits dominated by the (1011) and (1010) crystal faces generally manifest porous and loosely packed structures that consist of disordered hexagonal plates [117, 118, 119]. This specific morphology not only amplifies surface roughness and crystallographic heterogeneity but also aggravates hydrogen evolution and self‐corrosion due to the expanded interfacial area, as well as accounting for internal short circuits and the accumulation of orphaned inactive Zn during battery cycling [120, 121]. Consequently, the crystallographic orientation of metal electrodes fundamentally influences the morphology evolution and stability of Zn deposits during battery operation.

Moreover, the surface thermodynamic properties (e.g., surface energy and adsorption energy) of conductive hosts are equally critical in controlling Zn deposition. According to classical nucleation theory, the Δ*G*_nucleation_ is controlled by the surface free energy (Δ*G*_s_) and the bulk free energy (Δ*G*_v_). The relevant equations are given below:

(1) $$\Delta G=-\Delta G_{v}+\Delta G_{s}=-V\Delta g_{v}+S\gamma$$

where *V* is model volume, S is surface area, ∆g_v_ is the free energy change per unit volume, and parameter γ is the surface energy between the new phase and the environment. Therefore, the formation of a new solid phase at a heterogeneous interface requires extra thermodynamic costs surpassing the Δ*G*_nucleation_ [122]. The maximum Δ*G*_nucleation_ determines the critical nucleus sizes. Generally, the Δ*G*_nucleation_ is overcome by the applied electric field, which provides the necessary thermodynamic driving force for metallic cluster formation (Figure 3f) [123]. During galvanostatic deposition, the nucleation overpotential and plateau overpotential serve as quantitative metrics for evaluating the driving forces required for nucleation initiation and progressive growth (Figure 3g [124]). Chao et al. [125] established a Zn‐specific nucleation theory to clarify Zn electrodeposition in aqueous electrolytes and elucidated the relationship between Δ*G*_nucleation_, critical nucleus length (a^*^), and nucleation overpotential (*η*). Under overpotential (η)‐dominated deposition, the relevant equations are presented below:

(2) $$\begin{array}{c} \;a^{*}=\frac{2V_{m}}{\sqrt{3}\mathrm{zF}}\;\left(\frac{\gamma }{\eta }\right) \end{array}$$

(3) $$\begin{array}{c} \;\Delta G^{*}=\frac{2\sqrt{3}hV_{m}}{\mathrm{zF}}\;\left(\frac{\gamma^{2}}{\eta }\right) \end{array}$$

where z is the Zn charge, V_m_ is molar volume, and F is the Faraday constant. Therefore, the Δ*G*_nucleation_ and a^*^ are closely correlated with *η*. Meanwhile, *η* quantifies the thermodynamic energy necessary for the formation of critical atomic clusters, a parameter intrinsically linked to the surface thermodynamic properties of ZMAs, including surface energy, adsorption energy, and binding energy [126]. Furthermore, the plateau overpotential characterizes the equilibrium potential during subsequent Zn deposition phases [68, 127]. A conventional perspective holds that modulating the thermodynamic properties of the host, primarily by increasing adsorption and binding energies to lower nucleation overpotential, is key to the achievement of dense nucleation sites and a uniform deposition morphology [37, 57]. During the nucleation stage of Zn deposition, the crystallographic thermodynamics play a dominant role in the morphology evolution of Zn deposits at low current densities. Siegel et al. [122] established an ideal theoretical model to systematically correlate thermodynamic overpotential with the surface energy of distinct crystallographic planes. Crystal faces and edges characterized by high surface energy function as preferred sites for Zn^2+^ ion capture and preferential nucleation due to the diminished thermodynamic cost that reflected in their lower overpotential. Similarly, the surface energies of different crystal planes and their corresponding electrochemical activation current densities demonstrated an analogous trend, as illustrated in Figure 3h [51]. Theoretically, the thermodynamically favorable Zn plane is energetically favored to dominate the morphology evolution of Zn electrodeposits as the lowest surface energy across repeated plating/stripping processes [122]. Therefore, the thermodynamically favorable crystal plane with low energy barrier has dominated morphology evolution, thereby preferentially forming the hexagonal plates (also known as the Wulff construction) [128]. Nevertheless, the operational current densities in practical AZMBs far surpass the activation current densities of various crystal faces, thereby promoting multidirectional crystallization.

The thermodynamic stability of host materials in aqueous electrolytes also requires careful consideration. Firstly, the corrosion resistance of metallic hosts constitutes a vital consideration [129]. To illustrate, Al‐ or Ni‐based hosts are prone to corrosive degradation even in mildly acidic electrolytes, owing to their high electrochemical reactivity [130, 131]. Moreover, the onset of galvanic coupling between the host and ZMAs can markedly intensify undesirable side reactions, such as the HER and further corrosive processes [132, 133]. A typical example is the bimetallic Cu‐Zn system, where severe galvanic corrosion triggers pronounced HER on the Cu surface alongside persistent Zn dissolution [67]. Notably, spontaneous galvanic corrosion facilitates preferential dissolution at the Zn‐Cu interface owing to shortened electron pathways, which results in interfacial separation and shedding of active Zn. From the thermodynamic perspective, the negative ∆*G* will provide the driving force to move the electrons from Zn to Cu: [133]

(4) $$\begin{array}{ccc} \Delta G & = & -n\mathrm{EF}\left(E_{\mathrm{Cu}/Cu^{2+}}^{0}-E_{\mathrm{Zn}/Zn^{2+}}^{0}\right)\\ & = & -1\;\;\mathrm{mol}\times 96485\;\;C\;\;\mathrm{mo}l^{-1}\times \left(+0.34\;V-\left(-0.76\;V\right)\right)\\ & = & -10613.25\;J \end{array}$$

Conversely, host materials possessing a high HER overpotential exhibit high thermodynamic stability. For instance, a tin‐coated copper (Sn‐Cu) host demonstrated no visible gas evolution upon aging, attributable to the intrinsic HER‐inert character of metallic Sn. Furthermore, the Sn coating markedly reduced the corrosion current density to nearly negligible levels, indicating effective suppression of galvanic corrosion and consequently promoting the system toward a state of electrochemical stability. As an alternative strategy, the utilization of inert titanium (Ti) foil as a host confers an exceptionally high HER overpotential, thereby circumventing galvanic corrosion and associated parasitic reactions [38, 56].

### 2.2 The Kinetic Bottlenecks for ZMAs

**FIGURE 4:**
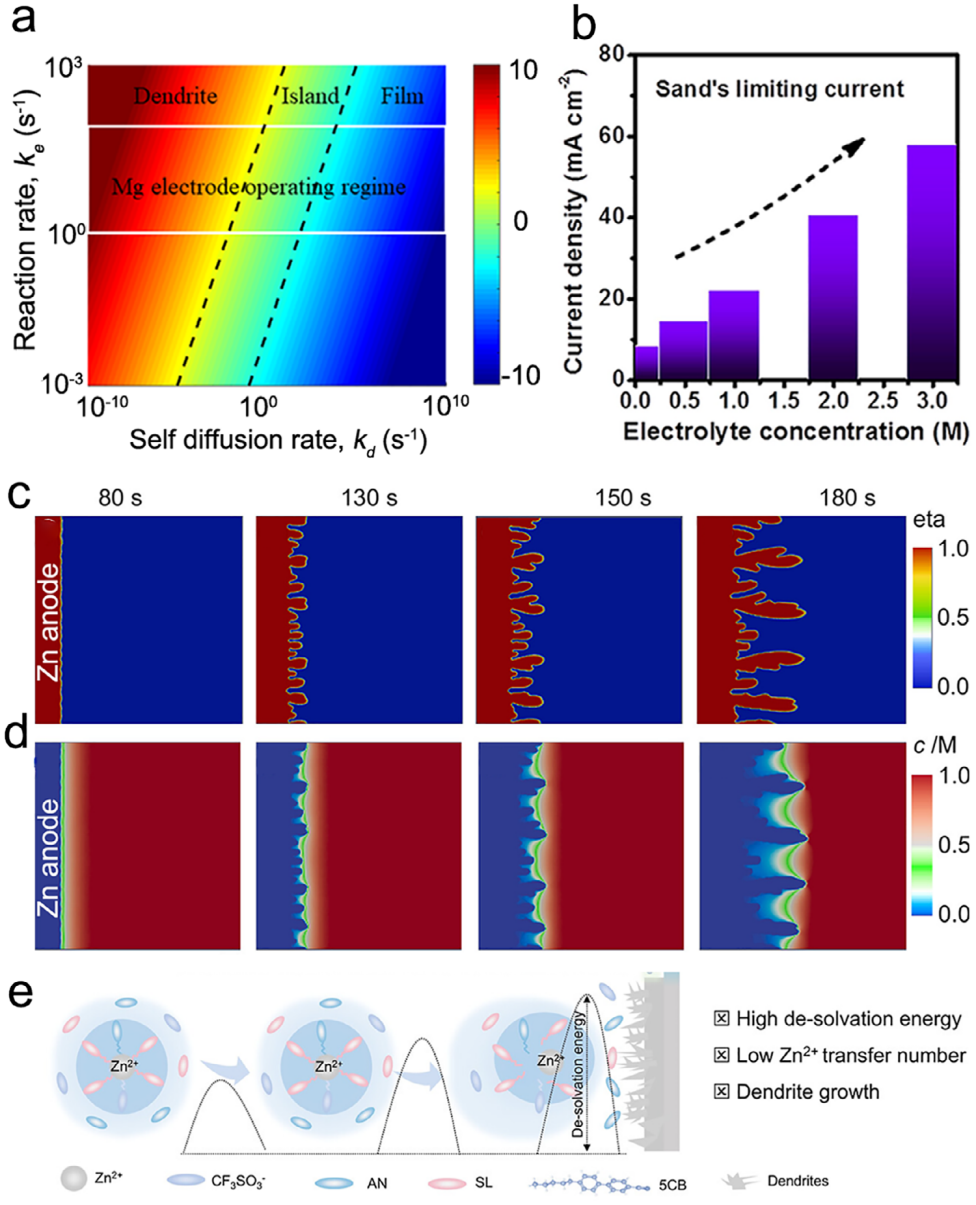
(a) Effects of reaction rate versus self‐diffusion rate on deposition morphology [137]. Copyright 2018, American Chemical Society. (b) The relationship between Sand's limiting current densities and electrolyte concentrations. (c) Electrode morphology evolution after Zn electrodeposition for 80s, 130s, 150s and 180s and (d) the corresponding Zn‐ion concentration evolution [143]. Copyright 2022, American Chemical Society. (e) Schematic illustration of the interfacial behavior in ANSL electrolytes [147]. Copyright 2025, American Chemical Society.

Beyond intrinsic thermodynamic considerations, comprehensive investigations have established that kinetic parameters (e.g., electrochemical reaction rates, ion diffusion rates, and desolvation) exert a predominant influence on the morphological evolution of Zn deposits [134, 135, 136, 137]. These kinetic processes are closely correlated with applied current density, electrolyte concentration, and the electric field distribution. The Damköhler number (Da = *J*_0_/*J*_lim_ or *k*_e_/*k*_d_) is used to quantify the relative rates of the electrode reaction and ion diffusion (Figure 4a) [43]. Phase‐field simulations further reveal that smooth deposition is favored under low current density conditions (Da << 1), whereas pronounced dendritic growth prevails under high current density conditions (Da >> 1). In addition, the classical Sand's time (*τ*) model illustrates an intimate dependence of Zn dendritic growth on the current densities in theory [55, 138, 139]. The corresponding equations can be described as follows: [140]

(5) $$\begin{array}{c} \tau =\pi D\frac{eC_{0}(\mu_{a}+\mu_{Zn^{2+}})}{2J\mu_{a}} \end{array}$$

where *τ* is the start time of Zn dendrite growth, *D* is the diffusion coefficient, *e* is the electronic charge, and *C*_0_ is the initial concentration of Zn^2+^ ions. *µ*_a_ and *µ*_Zn_^2+^ represent the anionic and Zn^2+^ ion mobility, respectively. In the light of this model, the high current density, low Zn^2+^ concentration, and restricted Zn^2+^ ion diffusion rate collectively contribute to the dendritic growth, a phenomenon directly attributed to undesirable kinetic processes [106]. In particular, rapid Zn^2+^ depletion at the electrode/electrolyte interface under high current densities forces the electrode reaction into a diffusion‐limited pattern, where the reaction rates substantially overwhelm diffusion rates. Under this pattern, the inadequate Zn ions supply induces severe concentration polarization and thus directly initiates the dendritic formation. Increasing the electrolyte concentration effectively mitigates concentration polarization, thereby inhibiting the transition of the electrode reaction into a diffusion‐controlled regime. As illustrated in Figure 4b, the diffusion‐limited current density exhibits a marked increase with elevated electrolyte concentration, thereby prolonging the *τ* for Zn deposition under high current densities [51, 55, 141].

Moreover, localized current density intensification at defects, cracks, and tips results in an inhomogeneous distribution of electric field, thereby leading to uneven nucleation and dendrite growth [142]. The phase‐field simulation results of Zn deposition revealed the process of Zn dendrite growth and the corresponding variation of electric field and concentration field (Figure 4c,d) [143]. During the electrodeposition process, the evolving morphology of Zn deposits continuously modifies the electrode geometry, which in turn dynamically changes the distribution of the electric field and alters the electrolyte concentration profile at the electrode‐electrolyte interface. In general, the intensified electric fields at high‐curvature tips of Zn dendrites promote the preferential migration and adsorption of Zn^2+^ ions [144]. Therefore, a fast reaction rate usually tends to occur on the tips of these protuberances because of a higher Zn^2+^ ion concentration than in other regions, accelerating the growth of Zn dendrites. Yufit et al. [145] have comprehensively studied the dissolution of dendritic Zn deposits via operando visualization and multi‐scale tomography techniques. Once the loosely packed dendritic networks form, they cannot be fully eliminated during dissolution owing to inferior Zn stripping reaction kinetics. A rapid dissolution rate at the Zn dendrite root was observed, characterized by the progressive thinning of dendritic trunks and their eventual detachment from the electrode surface, resulting in the formation of electrochemically inactive “dead Zn”. This phenomenon is attributed to the extended ion‐electron transport pathways across the deposits and sluggish ion diffusion kinetics [43]. The processes of tip growth and root dissolution create a self‐perpetuating cycle of dendritic regeneration, which not only accumulates electrochemically inactive Zn but also heightens short‐circuit susceptibility, thereby severely compromising the CE and cycling stability of ZMAs. In addition, the strong ion‐dipole interaction between Zn^2+^ and H_2_O in aqueous electrolytes contributes to the formation of stable solvation structures of Zn(H_2_O)_6_^2+^ [36, 146]. During the Zn deposition process, the desolvation energy barrier of hydrated Zn^2+^ needs to be overcome to break the Zn^2+^‐H_2_O bonds. Meanwhile, the desolvation of hydrated Zn^2+^ as a rate‐determining step of the charge transfer at the interface dominates ion diffusion and Zn nucleation. High desolvation energy barrier and low charge transfer number deteriorate the interfacial reaction kinetics and consequently accelerate the formation of Zn dendrites (Figure 4e) [147]. Moreover, the released water molecules during desolvation exhibit higher electrochemical activity than bulk water, which continuously corrodes the ZMAs and induces parasitic reactions and hydrogen evolution. Accordingly, accelerating the desolvation of hydrated Zn^2+^ is critical for developing high‐performance and long‐cycling AZMBs.

## 3 The Rational Design Strategies of Hosts for Advanced ZMAs

The widespread studies have provided compelling evidence that 3D host design offers a promising way to effectively manipulate the Zn deposition process through regulating the thermodynamic stability of ZMAs in aqueous electrolytes, interfacial reaction kinetics, and Zn^2+^ ion diffusion kinetics [74, 109, 148, 149]. In this section, the advanced design strategies of 3D hosts for improving the properties of ZMAs are categorized into surface modification, interface engineering, and spatial confinement. Specifically, the design principles of the aforementioned strategies are systematically clarified from thermodynamic and kinetic perspectives. Surface modification with zincophilic materials improves the adsorption energy of Zn^2+^ and lowers the 𝛥*G*_nucleation_, facilitating homogeneous Zn nucleation. The functional interfacial layers on the host surface can optimize interfacial reaction kinetics by redistributing interfacial electric fields, regulating local ion concentration, and accelerating desolvation, as well as enhancing the thermodynamic stability of ZMAs by electrolyte shielding [66, 150]. 3D hollow hosts with tunable pore sizes and ordered structures enable the gradient distribution of electric and concentration fields, and accelerate ion diffusion, thus achieving spatially confined deposition. Moreover, sufficient interior space of hosts can alleviate structure stress caused by volume expansion during the charging/discharging process [68, 134].

### 3.1 Surface Modification

Benefiting from prominent merits in homogenizing interfacial electric field, lowering local current density, and buffering volume expansion, 3D conductive hosts have been extensively employed to design high‐performance ZMAs [90]. Nevertheless, commercial 3D conductive hosts possess inferior adsorption capacity for Zn^2+^ ions due to the insufficient zincophilic sites and large lattice mismatch of Zn metal, which leads to high 𝛥*G*_nucleation_ and severely restricts homogeneous Zn nucleation [42, 151, 152]. Generally, the Zn adatoms spontaneously aggregate into isolated Zn crystallites on the host surface due to an excessively high heterogeneous Δ*G*_nucleation_, and thus form an uneven interfacial electric field distribution to trigger subsequent dendritic growth [153]. Anchoring abundant metallic or non‐metallic zincophilic species on the host surface as one of the prevailing surface modification strategies has been widely utilized to enhance host zincophilicity [3, 56, 58]. Nowadays, numerous 3D conductive hosts with diverse zincophilic species, including N‐doped carbon [151], carbon defects [81], functional groups [105], and metal clusters [40], have been designed to reduce the Δ*G*_nucleation_ and achieve uniform Zn nucleation. Moreover, constructing a lattice‐matched surface of hosts for the Zn(002) crystal plane can reduce the thermodynamic energy barriers and establish the thermodynamically favorable pathway for oriented nucleation and growth [81, 154].

#### 3.1.1 3D Conductive Hosts with Non‐Metal Zincophilic Sites

**FIGURE 5:**
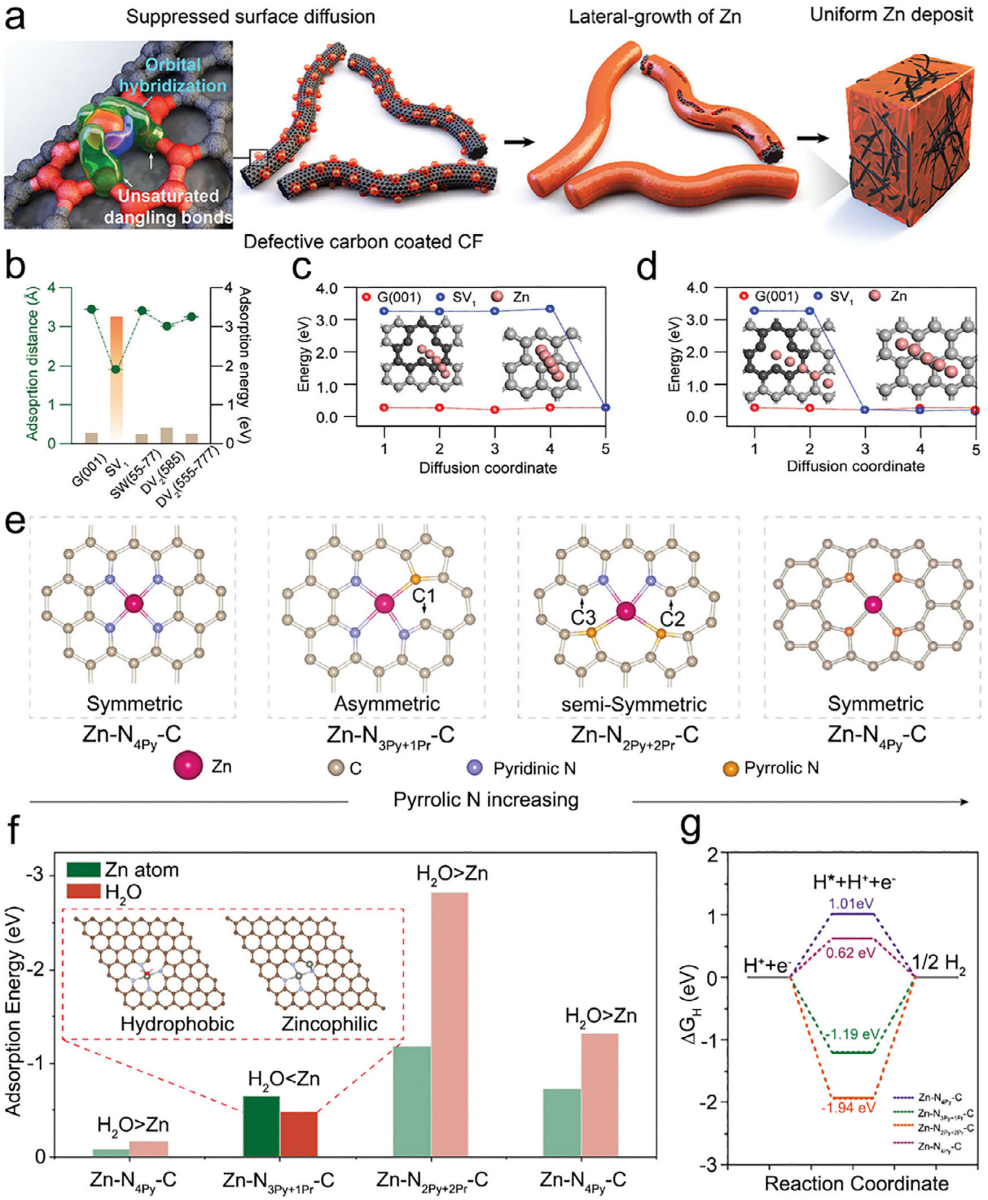
(a) Schematic illustration of Zn growth principles on the defective carbon layer‐coated 3D carbon felt. (b) Comparison of adsorption energies for Zn adatoms among the G (001) and various types of point defects. (c, d) Changes in the energy profiles of Zn on the SV_1_ defect‐containing carbon surface with movement of the adsorbed Zn atom crossing the C‐C bond and along the C‐C bond, respectively [81]. Copyright 2020, Royal Society of Chemistry. (e) Atomic structures of Zn‐N_4_‐Cs. (f) The adsorption energies of H_2_O molecule and Zn atom on the Zn‐N_4_‐Cs. (g) the calculated H* free energies on the Zn‐N_4_‐Cs [155]. Copyright 2025, Wiley‐VCH.

Directly engineering non‐metal zincophilic sites (e.g., nitrogen (N)/oxygen (O) functional groups and defects) on 3D conductive hosts is an effective strategy to prominently boost the zincophilicity of hosts via enhancing the adsorption energy toward Zn^2+^ ions. For instance, Lee et al. [81] have reported zincophilic 3D conductive hosts by introducing single‐vacancy carbon defects on the carbon felt. Of note, the carbon defects endowed the hosts with strong adsorption energy for Zn deposits through forming interatomic orbital hybridization, consequently achieving uniform Zn deposition. As shown in Figure 5a, a defective carbon surface accompanied by an exceptionally strong orbital hybridization with Zn metal effectively enhanced the Zn^2+^ ions capturing capability of hosts, thus inducing uniform Zn nucleation. On the contrary, randomly oriented Zn nuclei agglomerated on the carbon felt without defects due to the low zincophilicity. Then, the Zn adatoms diffused along the surface and grew on the tips of the deposited Zn, thus leading to an uneven deposition morphology. In the initial stage of nucleation, the carbon defects as the zincophilic sites effectively facilitated the homogeneous nucleation, accordingly generating a uniform Zn deposit on the surface. With the increase of deposition time, the smooth Zn coated the whole carbon fiber and then grew laterally to connect contiguous fibers. In order to understand the connections between the hosts and Zn deposits, the interatomic interaction was explored via the DFT calculation. Obviously, the single vacancy defects (SV_1_) provided stronger adsorption energy for Zn atoms compared with other defects and graphene owing to the strong orbital hybridization (Figure 5b). Moreover, the diffusion energy barrier of Zn adatoms on the SV_1_ was much higher than that of Zn and graphene, which further demonstrates a significantly suppressed 2D diffusion of Zn adatoms (Figure 5c,d). Consequently, the strong interaction between the defect‐modified 3D CF and metallic Zn effectively suppressed Zn dendrite growth and enabled a prolonged cycling life with low voltage hysteresis for the corresponding Zn‐bromine flow batteries. Moreover, Cao et al. [105] have proved that in situ integrating the N‐doped vertical graphene (VG) nanosheets on carbon cloth (N‐VG@CC) achieved a greatly enhanced zincophilicity of the 3D conductive host. The DFT calculation results revealed that the binding energy between N‐containing functional groups and Zn atoms was higher than that of the graphene, proving a strong interaction between the N‐VG and metallic Zn. Benefiting from the strong interaction, the N‐VG@CC delivered the reduced nucleation overpotential and high CE, endowing the Zn@NVG@CC electrode with long cycle stability and low voltage hysteresis. Qiao et al. [151] further systematically studied the functional mechanism of nitrogen nucleation sites in the aspect of manipulation of Zn deposition through advanced in situ/ex situ techniques. The results of near‐edge X‐ray absorption fine structure (NEXAFS) and in situ Raman spectra revealed that the Zn─N bonds between the Zn^2+^ ions and pyridine promoted homogeneous nucleation on carbon hosts, ultimately inducing uniform Zn deposition. As expected, carbon hollow spheres with zincophilic nitrogen sites (CnC HS) exhibited a more stable reversibility, ensuring the as‐prepared ZMAs with boosted electrochemical performance. From an in‐depth fundamental insight, Yang et al. [155] explored Zn‐N_3Py+1Pr_‐C hosts featuring the zincophobic/hydrophilic nature through reconfiguring electronic interactions between Zn atoms and coordinating functional groups, including pyridinic nitrogen (Py‐N) and pyrrolic nitrogen (Pr‐N), on single‐atomic Zn‐N_4_‐C. Varied Zn‐N_4_‐C structures, including Zn‐N_4Py_‐C, Zn‐N_3Py+1Pr_‐C, Zn‐N_2Py+2Pr_‐C, and Zn‐N_4Pr_‐C, were constructed by regulating coordinated Pr‐N and Py‐N numbers (Figure 5e). Among them, the C_1_ and Pr‐N atoms in the asymmetric Zn‐N_3Py+1Pr_‐C host provided strong bonding with Zn atoms due to a redistribution of electronic states derived from Zn 3d band center downshifting and a localized nonbonding state in the N/C 2p bands. Benefiting from a unique coordination structure, the Zn‐N_3Py+1Pr_‐C host provided a stronger adsorption energy for Zn atoms than that for H_2_O, leading to a preferential selectivity toward Zn atom adsorption (Figure 5f). Besides, the high HER energy barrier on the Zn‐N_3Py+1Pr_‐C host demonstrated a potential hydrophobic nature (Figure 5g). Consequently, the asymmetric ZnN_3Py+1Pr_‐C host delivered a superior CE of 98.3% over 500 cycles, ensuring a robust long‐cycle life over 500 h even at a high depth of discharge (DOD) of 50% in a Zn‐N_3Py+1Pr_‐C@Zn||Zn‐N_3Py+1Pr_‐C@Zn symmetrical battery.

#### 3.1.2 3D Conductive Hosts with Metal Zincophilic Sites

**FIGURE 6:**
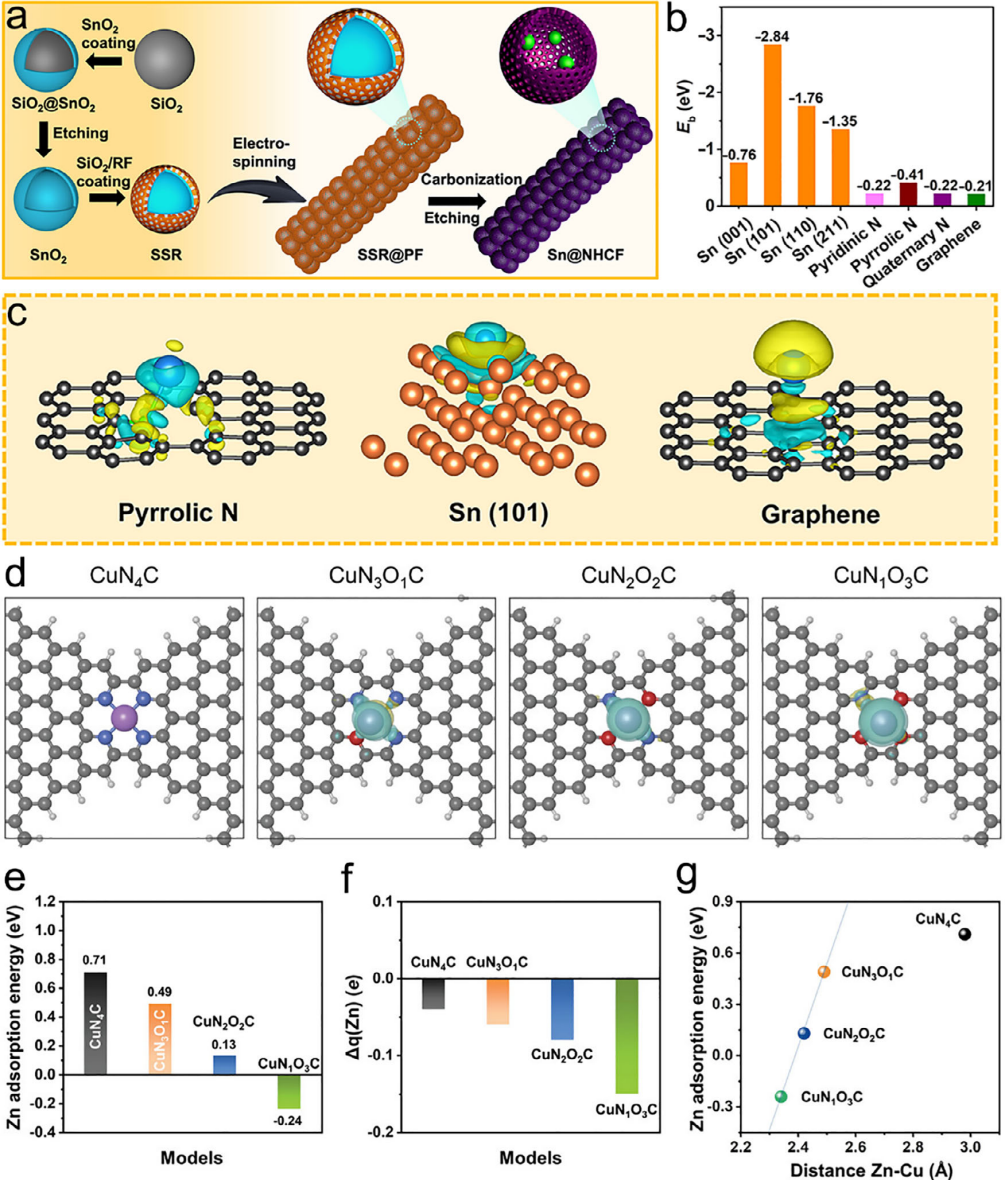
(a) Schematic illustration of the synthesis of Sn@NHCF. (b) Interfacial charge‐density models of pyrrolic N, Sn (101), and graphene. (c) Interfacial charge‐density models of pyrrolic N, Sn (101), and graphene [84]. Copyright 2022, American Association for the Advancement of Science. (d) The atomic configurations and charge‐density distribution of different models. (e) The adsorption energies between the models and Zn atoms. (f) Bader charge difference before and after Zn adsorption. (g) The variation of Zn‐Cu atomic distance with the Zn adsorption energies [158]. Copyright 2024, Wiley‐VCH.

The incorporation of metal‐based zincophilic sites (e.g., Ag [57], Sb [156], Bi [157], Zn [89], Cu [158], Sn [98], etc.) into 3D conductive hosts has emerged as a pivotal strategy for further strengthening Zn^2+^ adsorption energy, aiming to induce a more uniform deposition morphology. For instance, Yu et al. [84] integrated the N‐doped hollow carbon spheres with Sn nanoparticles on the inner surface of 3D hollow fibers (denoted as Sn@NHCF) through hard template methods as a zincophilic host for achieving high‐performance ZMAs (Figure 6a). The DFT calculation results revealed that the Sn zincophilic sites provided the strongest binding energies for Zn atoms, which are much higher than those of pyrrolic N and graphite (Figure 6b). Moreover, the enhanced charge transfer between Sn and Zn atoms further confirmed that the zincophilic Sn nanoparticles offered strong interaction for metallic Zn, thus reducing the 𝛥*G*_nucleation_ (Figure 6c). The Sn@NHCF host exhibited the lowest nucleation overpotential among various types of hosts, which is in accordance with the DFT calculations. Moreover, the low electrocatalytic activity of Sn metal enabled the Sn@NHCF host to exhibit an enhanced overpotential of HER compared to the NHCF host. Benefiting from the above superiorities, a preferential Zn nucleation occurred on the Sn particles within the hollow carbon spheres, and the subsequent deposition of metallic Zn was expected to be constrained inside the hollow fibers. With the increased Zn deposition capacity, the deposited Zn covered both the inner and exterior surfaces of fibers and then filled the porous network without dendrites. On the contrary, numerous Zn dendrites formed on the surface of Zn foils due to the low zincophilicity. As expected, the Sn@NHCF host exhibited low overpotential and high CE, which endows the Sn@NHCF‐Zn electrode with prolonged cycling lifespan and low voltage hysteresis in a symmetric battery. Besides, Yin et al. [159] have proposed the Sn layer‐coated 3D carbon felt host (SH) for Zn deposition, fabricated by facile magnetron sputtering. DFT calculations and experimental results also demonstrated that a metallic Sn layer rendered a significantly enhanced interaction between the hosts and Zn metal. Clearly, the Zn deposits covered the surface of Sn and then connected to those on the other fibers without obvious Zn dendrites. Benefiting from potential merits, the as‐fabricated SH showed low Zn nucleation overpotential, thus ensuring the corresponding ZMAs with a long cycle life with high CE. Beyond that, Wang et al. [160] prepared the 3D carbon framework with a trace amount of metallic Zn^0^ evenly distributed on the surface as the zincophilic hosts through ZIF‐8 treated with an optimized temperature. The even‐distributed Zn^0^ has played a dominant role in inducing uniform nucleation while providing a simultaneously high overpotential for HER, thus forming a smooth and dense deposition morphology without Zn dendrites. Similarly, Wang et al. [18] fabricated a bacterial cellulose‐derived 3D carbon nanofiber network with well‐dispersed Zn seeds (CNF‐Zn), to be used as the host for preparation of dendrite‐free ZMAs. Trace amounts of metallic Zn in CNF‐Zn host function as nucleation sites, significantly reducing the nucleation overpotential and enabling a highly reversible deposition/stripping process. As a result, the 3D CNF‐Zn host endowed the corresponding CNF‐Zn@Zn anode with a high CE and a remarkable cycling stability in the CNF‐Zn@Zn//NaV_3_O_8_·1.5H_2_O full battery. In addition, Tao et al. [104] developed leaf‐like Zn‐coordinated zeolitic imidazolate framework (ZIF‐L) nanoflakes on Ti mesh with atomically dispersed Cu on the surface as the zincophilic host (CuZIF‐L@TM) for the dendrite‐free ZMAs. The experimental data and theoretical calculations confirmed that the atomically dispersed Cu as the nucleation sites provided a much higher adsorption energy for metallic Zn than the Zn–Zn interaction. This strong adsorption of Cu not only decreased the nucleation barrier but also improved the Zn migration barrier between adjacent adsorption sites, effectively restricting 2D Zn atom diffusion on the surface. Owing to the high zincophilicity, the smooth Zn nanoplates grew along the CuZIF‐L nanoflakes without dendrites. As expected, the CuZIF‐L hosts delivered a high CE and low overpotential, endowing the corresponding symmetric cell with a stable operation over 1100 h with low voltage hysteresis.

Furthermore, atomically dispersed metallic zincophilic sites integrated on the host surface can further optimize the Zn deposition behaviors. For instance, Lee et al. [158] developed a highly zincophilic host featuring abundant nucleation sites for uniform Zn nucleation via incorporating a well‐dispersed Cu single atom within an N, O co‐doped porous carbon framework (CuNOC). The calculations indicated that the coordinated O atoms surrounding the Cu atoms not only attenuated the bonding strength between the Cu atom and the neighboring atoms but also reinforced the interaction with adsorbed Zn atoms, accounting for markedly enhanced charge transfer between CuNOC and Zn atoms (Figure 6d). Compared to CuN_4_C, CuN_1_O_3_C achieved an improved adsorption energy (−0.24 eV) for Zn atoms due to the co‐doped O atoms, pronouncedly enhancing the host zincophilicity (Figure 6e). Moreover, the calculated charge transfer values based on the Bader charge model, denoted as electron donation from Zn atoms for all of atomic configuration models, were correlated well with the Zn adsorption energy, further demonstrating an enhancement of electronic interaction at the CuNOC‐Zn interface (Figure 6f). All of the CuNOC hosts delivered markedly shortened Zn‐Cu distances compared to CuN_4_C, along with a negative correlation with Zn adsorption energy, conclusively highlighting the essentiality of N, O co‐doping in promoting the zincophilicity (Figure 6g). Leveraging the aforementioned benefits, the composite CuN_4_C/Zn anode achieved dendrite‐free morphology and high reversibility of the plating/stripping process over 1000 cycles at 1mAh cm^−2^, ensuring stable operation over 400 cycles at 0.5 A g^−1^ in a CuN_4_C/Zn||V_2_O_5_ full cell. Beyond zincophilicity, the chemical stability and catalytic activity toward HER have emerged as critical screening parameters for the assessment of Zn alloy anodes. Zhao et al. [60] prepared a stable Bi@Zn anode with the potential advantages of thermodynamic inertia and kinetic zincophilia through a comprehensive screening of hetero metals. Theoretical calculations indicated that Bi metal showed the highest adsorption energy for Zn atoms and the lowest adsorption energy (*ΔG*_H*_) for adsorbed H atoms among all of the heterogeneous metals, including Bi, Zn, Fe, Co, Ni, Sn, and Cu, indicating the intrinsic natures of superior zincophilicity and robust chemical stability in the electrolytes. Hence, the Zn‐Bi alloy manifested low nucleation overpotential of Zn plating, high HER overpotential, and outstanding anticorrosion performance, successfully achieving dendrite‐free Zn deposits while efficiently inhibiting detrimental parasitic reactions. The corresponding Bi@Zn anode delivered stable operation with a small voltage hysteresis of≈55 mV over 4700 cycles at 10 mA cm^−2^ [60].

#### 3.1.3 3D Conductive Hosts with Alloy Seeds

**FIGURE 7:**
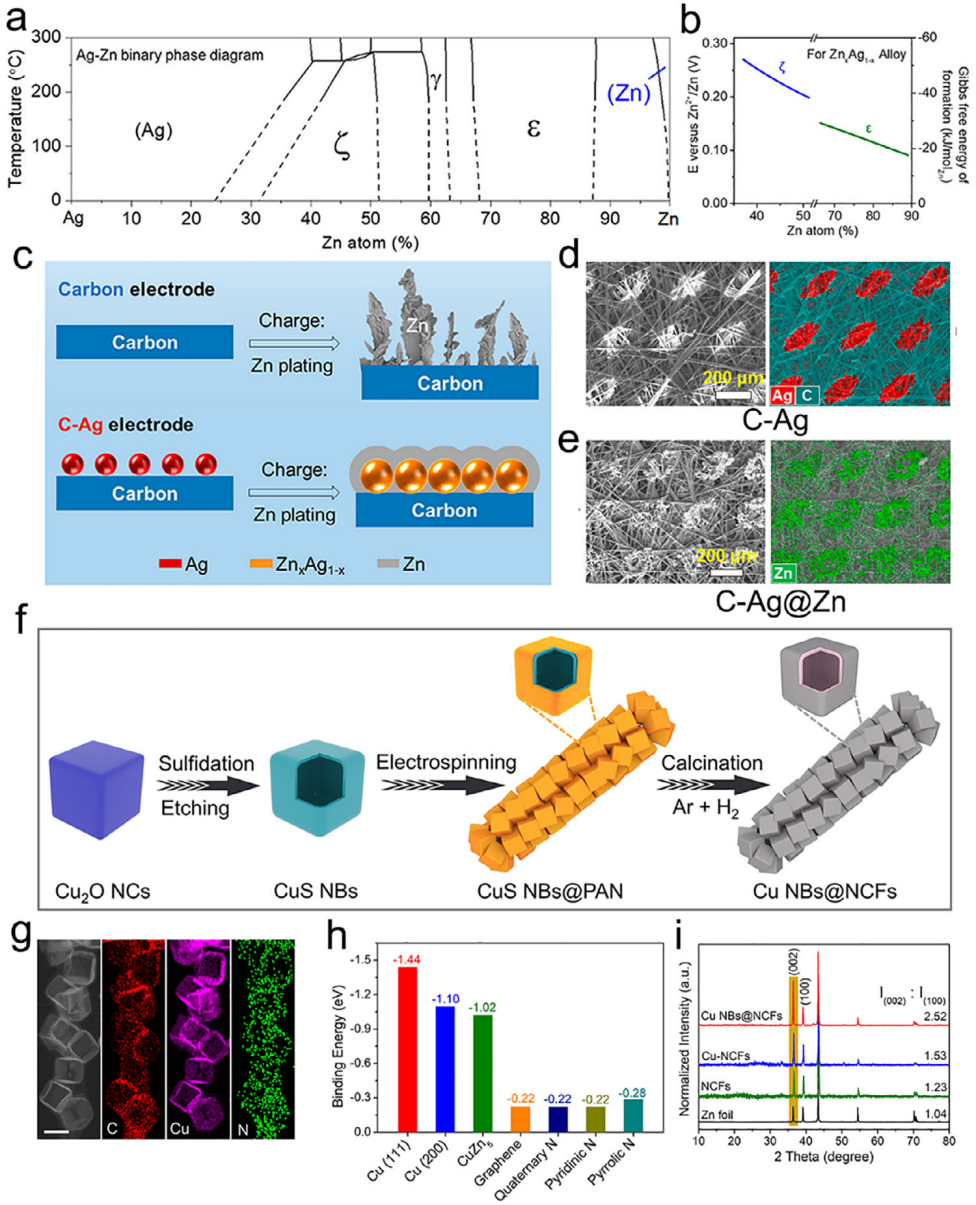
(a) Phase diagram of Zn with Ag. (b) Calculated Gibbs free energy of formation of alloy phases and the corresponding electrochemical potential shift of Zn^2+^/Zn*_x_* Ag_1‐_ *_x_* compared with that of Zn^2+/^Zn. (c) Schematic of Zn deposition on different hosts. SEM images and elemental mapping images of the C‐Ag (d) before and (e) after Zn plating [66]. Copyright 2021, American Chemical Society. (f) Schematic illustration of the synthetic process of Cu NBs@NCFs. (g) High‐angle annular dark‐field STEM and elemental mapping images of Cu NBs@NCFs. (h) Summary of the calculated binding energy of Zn atoms with different hosts. (i) XRD patterns of the Cu NBs@NCFs host [162]. Copyright 2022, Wiley‐VCH.

Specifically, engineering zincophilic alloy seeds on the host surface has been applied to reinforce the interfacial interaction between Zn^2+^ and the host by forming solid solutions or intermetallic compounds [66, 76, 104, 161]. The Zn‐soluble materials such as Ag [66], Cu [162], Sn [76] usually present low 𝛥*G*_nucleation_ for Zn deposition owing to the formation of alloy interface layer. As shown in Figure 7a, the binary phase diagrams of Zn‐Ag alloys revealed that Ag can react with Zn to form crystalline ζ‐, γ‐, and ε‐Zn*_x_* Ag_1‐_ *_x_* alloy phases [66]. Besides, all of the Zn*_x_* Ag_1‐_ *_x_* alloy phases possess a negative Gibbs free energy of formation, which means the spontaneous formation of Zn*_x_* Ag_1‐_ *_x_* during Zn deposition (Figure 7b). In this way, Zn‐Ni, Zn‐Cu, Zn‐Sn, Zn‐Ag, Zn‐Se alloys featuring particular characteristics of crystalline phase have been utilized for minimizing 𝛥*G*_nucleation_ and inducing the homogeneous Zn deposition. For instance, Zhang et al. [66] achieved a high‐performance Zn‐Ag alloy anode by introducing heterogeneous Ag seeds on carbon paper (C‐Ag). Besides, the underlying formation mechanism of Zn Alloy seeds has been analyzed in detail. Clearly, the Ag seeds accommodated Zn by forming Zn*_x_* Ag_1‐_ *_x_* alloy phases, thus inducing the subsequent deposition of Zn with smooth morphology (Figure 7c). On the contrary, dendritic morphology was formed on the carbon fibers during the Zn deposition process. Furthermore, the elemental mapping images further proved the preferential Zn deposition on the Ag islands (Figure 7d,e). Consequently, the C‐Ag electrode delivered a negligible nucleation overpotential together with high CE, which endows the Zn‐Ag alloy anode with superior cycling lifespan. Besides, Chen et al. [150] also demonstrated that the Ag nanoparticles could work as heterometallic seeds for Zn deposition by forming the Zn‐Ag alloy. The DFT calculations revealed that the Ag and the Zn‐Ag alloy provided a higher binding energy for Zn than carbon. Besides, Zhang et al. [163] have demonstrated that the Cu mesh with electrical conductivity significantly decreased the energy barrier of Zn nucleation via a formative Cu‐Zn solid solution interface. Compared with the brass and graphene nanoribbons, the stronger binding energy existed between the Cu mesh and Zn deposits. Moreover, the Cu‐Zn solid solution formed in initial nucleation strengthened the binding energy to a lower stage. Finally, the Cu mesh showed a low overpotential and greatly improved the reversibility of Zn deposition owing to the above strong interaction. Furthermore, Lou et al. [162] developed a 3D hollow conductive host by embedding the Cu nanoboxes into N‐doped carbon fibers (denoted as Cu NBs@NCFs) via integrating hard‐templating with multi‐step annealing methods, achieving a uniform and dense Zn deposition (Figure 7f). The elemental mapping images of Cu NBs@NCFs revealed that Cu, C, and N elements were uniformly distributed throughout the hierarchical fibers, facilitating homogeneous nucleation (Figure 7g). Remarkably, a trace amount of CuZn_5_ solid‐solution formed on the surface of Cu NBs@NCFs during the Zn nucleation process. The zincophilic Cu and in situ formed Cu‐Zn solid solution minimized 𝛥*G*_nucleation_, thereby acquiring the low Zn nucleation overpotential. The DFT calculation was applied to investigate interatomic interactions between each component in Cu NBs@NCFs and metallic Zn. Clearly, the DFT calculation results revealed that the binding energy between the Zn atoms and the Cu or CuZn_5_ is higher than that of the graphene or N‐doped carbons (Figure 7h). According to the XRD patterns of Cu NBs@NCFs‐Zn, the improved intensity ratio of the Zn(002) and Zn(100) planes proved that the Cu NBs@NCFs efficiently exposed more metallic Zn(002) crystal planes, realizing the crystal orientation growth of ZMAs (Figure 7i). As a result, the Zn deposits initially covered the whole surface of Cu NBs@NCFs and then connected to the other fibers, finally forming uniform and compact Zn deposition. Consequently, the Cu NBs@NCFs host displayed a low overpotential and superior reversibility for Zn deposition/dissolution, endowing the Cu NBs@NCFs‐Zn electrode with a long‐term cycling lifespan without obvious Zn dendrites.

#### 3.1.4 Guiding Epitaxial Electrodeposition

**FIGURE 8:**
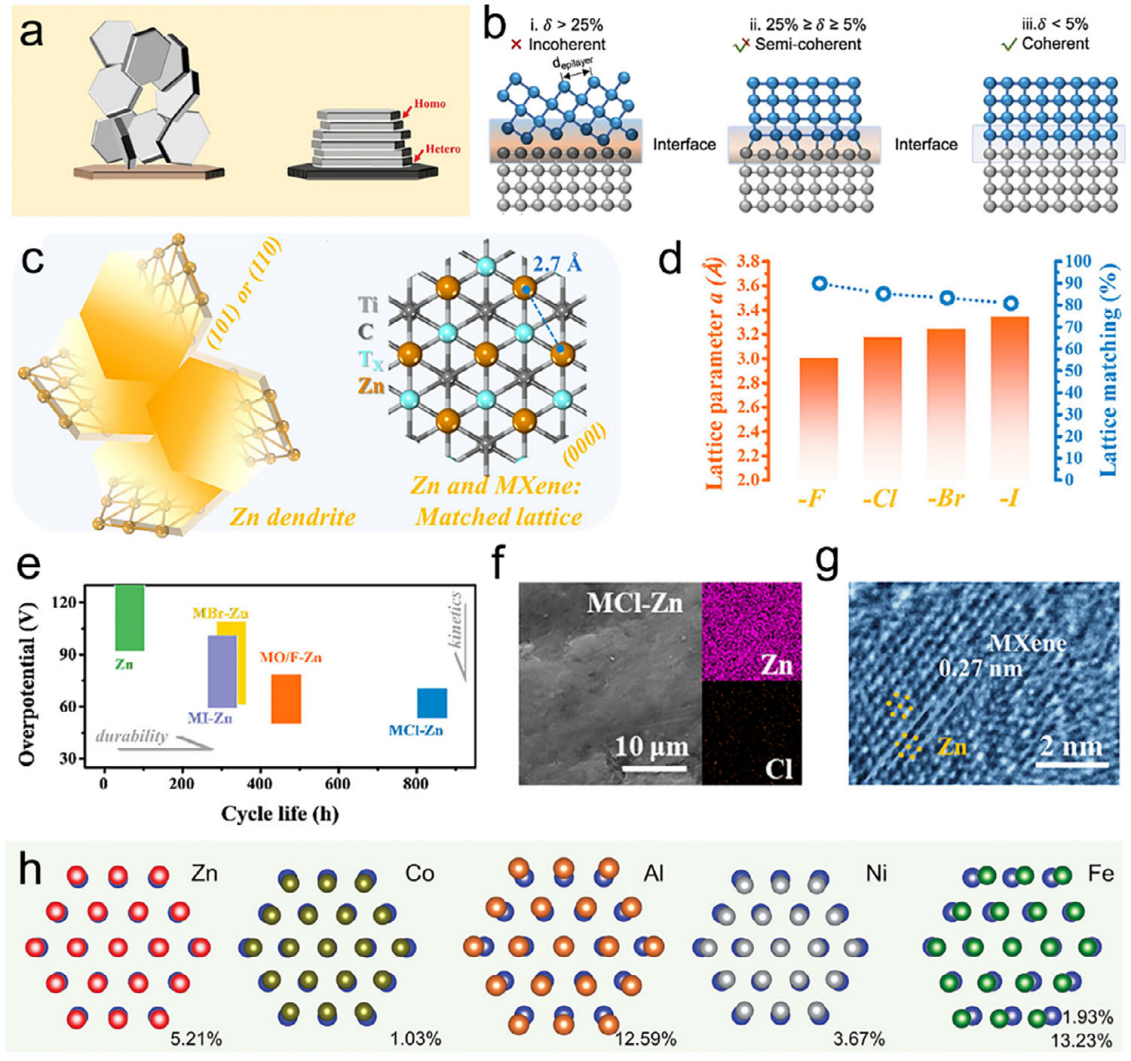
(a) Scheme illustrating the design principle of epitaxial metal electrodeposition [153]. Copyright 2019, American Association for the Advancement of Science. (b) Correlation between lattice strain and orientation of Zn electrodeposits on host surface [88]. Copyright 2025, Springer Nature. (c) Schematic illustration of Zn dendrite growth and MXene crystal structure matching Zn deposits on MXene. (d) Lattice parameters *a vs*. calculated lattice matching ratios of halogen‐terminated Ti_3_C_2_(F/Cl/Br/I)_2_. (e) Comparison of cycle durability and overpotential of symmetric batteries at 2 mA cm^−2^. (f) SEM images and EDX mapping of the MCl‐Zn electrode after cycling with a flat and smooth surface. (g) HRTEM image of the heterointerface region with the coexistence of the Ti_3_C_2_Cl_2_ matrix and Zn deposits [189]. Copyright 2021, American Chemical Society. (h) Epitaxial electrodeposition models for metals on Cu(111) plane [173]. Copyright 2023, American Chemical Society.

Crystallographic orientational texturing during Zn electrodeposition serves as a practical route to achieve homogeneous and thermodynamically stable electrodeposits that are critical for achieving prolonged lifespan of ZMAs [164]. To date, the Zn(002) crystallographic plane featuring a low surface energy, high atomic packing density, preferential lateral growth tendency, superior chemical stability, and remarkable electrochemical reversibility is recognized as a pivotal orientation for enabling dendrite‐free and long‐lasting ZMAs [112, 165]. From a thermodynamic perspective, the core of the heteroepitaxial strategy lies in constructing a lattice‐matched interface to establish a thermodynamically favored pathway for the nucleation and growth of the Zn(002) plane [85, 166, 167]. This directed control is enabled by the orchestrated coordination of two underlying thermodynamic mechanisms. The first mechanism involves lowering the nucleation barrier of the Zn(002) plane to guide oriented nucleation while simultaneously reducing the interfacial energy to stabilize the epitaxial interface [117, 128]. The second provides a flat and coherent Zn seed layer at the initial epitaxial interface, thereby maintaining a persistent energy minimization pathway that induces the subsequent preferential growth of the (002) plane [120, 167]. These mechanisms collectively establish the thermodynamic preference for the desired crystallographic orientation. In 2019, Archer and coworkers reported a pioneering electrochemical heteroepitaxy strategy for Zn electrodeposition, achieving preferentially oriented Zn(002) deposition (Figure 8a) [43]. Clearly, the minimal lattice mismatch between the graphene and the Zn(002) plane facilitated parallel Zn deposition via a heteroepitaxial growth mode. The favorable crystal symmetry and well‐matched lattice parameters for Zn crystallites further promoted the oriented deposition on the graphene surface. Moreover, even after the graphene surface is fully covered by the initial Zn layer, subsequent deposition proceeds via homoepitaxial growth, maintaining the (002) texture. As a result, the uniform and compact ZMAs dominated by the (002) texture demonstrated exceptional cycling reversibility over thousands of cycles across various current densities. Based on the lattice mismatch (*δ*) between the Zn electrodeposits and the hosts, the interfacial structure can be categorized into three types: incoherent (*δ* ≥ 25%), semi‐coherent (25% ≥ *δ* ≥ 5%), and coherent (*δ* ≤ 5%, Figure 8b) [88]. A lower lattice mismatch generally leads to a reduced lattice strain in the deposited Zn and a stronger crystallographic orientation relationship with the hosts. Therefore, identifying suitable substrates with minimal lattice mismatch to the Zn(002) plane is essential. Guided by the aforementioned screening principles, a variety of nanomaterials (e.g., graphene and the corresponding derivatives [57, 87], MXene [22, 45, 168], alloys [113, 118, 165], silicates [88]) have subsequently been explored as heteroepitaxial hosts to promote the predominant formation of (002)‐textured Zn deposits. The N‐doped graphene and N, O co‐doped graphene skins function as highly effective heteroepitaxial interphases that enable superior crystallographic alignment and surface planarity [19]. For instance, Xu et al. [169] designed a flower‐shaped carbon (C_flower_) host with microcrystalline graphitic layers, which could induce Zn deposition into an epitaxial growth pattern. The C_flower_ host has successfully induced Zn growth along the surface of graphitic layers by modifying the zincophilic oxygen/nitrogen dopants, thus hindering the formation of Zn dendrites. As expected, the C_flower_ host expressed a high CE, and the corresponding composite ZMAs achieved a long cycling lifespan. Furthermore, Ti_3_C_2_T*_x_* MXene has been broadly utilized for orienting Zn deposition, benefiting from its innate properties including good Zn(002) lattice matching, high conductivity, large specific surface area, and abundant polar surface terminals for direct Zn^2+^ interaction, thus establishing a superior epitaxial environment intrinsically without requiring extra modification. For example, Zhi et al. [67] designed a series of MXenes with various halogen functional groups as the hosts to induce uniform Zn deposition by heteroepitaxial growth. The density functional theory (DFT) simulation and experimental characterization identified that the growth of the (101) or (110) crystal plane of Zn has an extreme tendency to motivate the formation of irregular dendritic morphology (Figure 8c). On the contrary, both Zn deposits and MXene belong to the same hexagonal close‐packed dense lattice structure and possess close lattice parameters. Notably, all the MXenes with different functional terminations (Ti_3_C_2_F_2_, Ti_3_C_2_Cl_2_, Ti_3_C_2_Br_2_, and Ti_3_C_2_I_2_ MXenes) showed high lattice matching ratios between 81% and 90% (Figure 8d). The high lattice matching between the (00l) crystal plane of the MXene matrix and the (002) crystal plane of Zn enabled heteroepitaxial nucleation and growth in a planar manner. Among them, Ti_3_C_2_Cl_2_ with the moderate adsorption and diffusion coefficients for Zn^2+^ ions showed lower ove rpotential and superior cycle stability (Figure 8e). Furthermore, the smooth Zn deposits entirely covered the surface of Ti_3_C_2_Cl_2_ without any dendrites, while the buried MXene interface layer was hardly detected (Figure 8f). Moreover, the high‐resolution transmission electron microscopy (HRTEM) images showed that the protruding Zn atoms with an incompletely hexagonal arrangement structure on the surface of Ti_3_C_2_Cl_2_ prove an active regulation of MXene for the deposition path of Zn^2+^ ions (Figure 8g). In summary, Zn atoms constructed a coherent heterogeneous interface on the highly lattice‐matched MXene host, guiding the growth of Zn with the (002) crystal plane. Then, the initial Zn deposits served as a seed layer to induce the subsequent homogeneous Zn deposition and eliminate the physical basis of undesired dendrite growth. Compared with bare Zn metal, the MXene‐Zn anodes displayed higher reversibility and lower polarization, endowing the corresponding symmetric battery and full battery with long cycle stability even at high current density. Furthermore, a range of graphene‐like materials, including carbon dots (CDs) [41], boron nitride (BN) [56, 170], molybdenum disulfide (MoS_2_) [171], and graphitic carbon nitride (g‐C_3_N_4_) [23, 172], have been explored as effective heteroepitaxial hosts.

Equally significant, metal‐based hosts can facilitate the epitaxial deposition of metal anodes by exploiting crystallographic coherence between close‐packed planes [113, 166]. This principle is exemplified by the work of Chen et al. [173], who achieved epitaxial and non‐dendritic growth of various metal anodes (Zn, Co, Al, Ni, Fe) encompassing hexagonal close‐packed (hcp), face‐centered cubic (fcc), and body‐centered cubic (bcc) structures on single‐crystal Cu(111) hosts. The Cu(111) surface exhibits minimal lattice mismatch (<25%) and a high epitaxial driving force toward the close‐packed planes of these metal anodes, thereby dictating a crystallographically locked orientation throughout deposition (Figure 8h). Consequently, the metallic deposits formed a parallel alignment of their close‐packed planes with the Cu host. Multimodal crystallographic investigations verified that electrodeposits epitaxially grow along these close‐packed orientations, yielding planar and dendrite‐free anodes with a 100% preferred crystallographic alignment. Moreover, Zeng et al. [17] revealed that the single‐crystal Cu(100) provided an exceptional lattice coherence with the hexagonal close‐packed Zn(002) plane, achieving the Zn (002)‐textured deposits via the underpotential deposition. Moreover, Zhou et al. [118] certified that the high lattice mismatch of Zn‐Ag solid solution and Zn metal originating from their almost identical crystal structure significantly reduced the 𝛥*G*_nucleation_ of Zn deposition, thus establishing a Zn(002) crystal plane oriented growth mode and inherently suppressing dendrite formation. Recently, Zhang et al. [113] developed an Al‐0.1wt%Si host with negligible lattice spacing deviation, which represents an ultralow lattice mismatch (0.51%) with the Zn(002) plane and enables a Zn(002)‐textured nucleation process and uniform plating layer. The Al‐0.1wt%Si‐Zn (ASZ) anode exhibited an ultralong cycling lifespan over 8000 h at 5 mA cm^−2^ in ASZ||ASZ cells, guaranteeing the ASZ||MnO_2_ coin cells a long‐cycle life for more than 1500 cycles. To date, several alloy phases, such as ZnAl [113], ZnCu [50, 116, 174], ZnAg [118], ZnSn [175], ZnIn [176], and ZnSe [177], exhibit low lattice mismatch with Zn(002), which have been proved to achieve horizontal Zn deposition through heteroepitaxial growth.

### 3.2 Interface Engineering

Surface modification strategies enable homogeneous Zn nucleation on the host surface by regulating surface thermodynamic properties. The modulation of interfacial electric field distribution, interfacial ion flux, and Zn^2+^ ion desolvation via interface engineering can optimize interfacial reaction kinetics and thus guide the subsequent growth of dendrite‐free ZMAs [45, 59, 103]. Specifically, structure modulation of 3D zincophilic hosts has been widely utilized to homogenize the interfacial electric field, decrease the local current density, and optimize interfacial ion flux, thereby reconstructing the interfacial microenvironment [120, 170]. Moreover, building a functional interface layer on the host surface can both accelerate Zn^2+^ ion desolvation and suppress water‐related side reactions.

#### 3.2.1 Regulating the Interfacial Electric Field and Ion Flux

**FIGURE 9:**
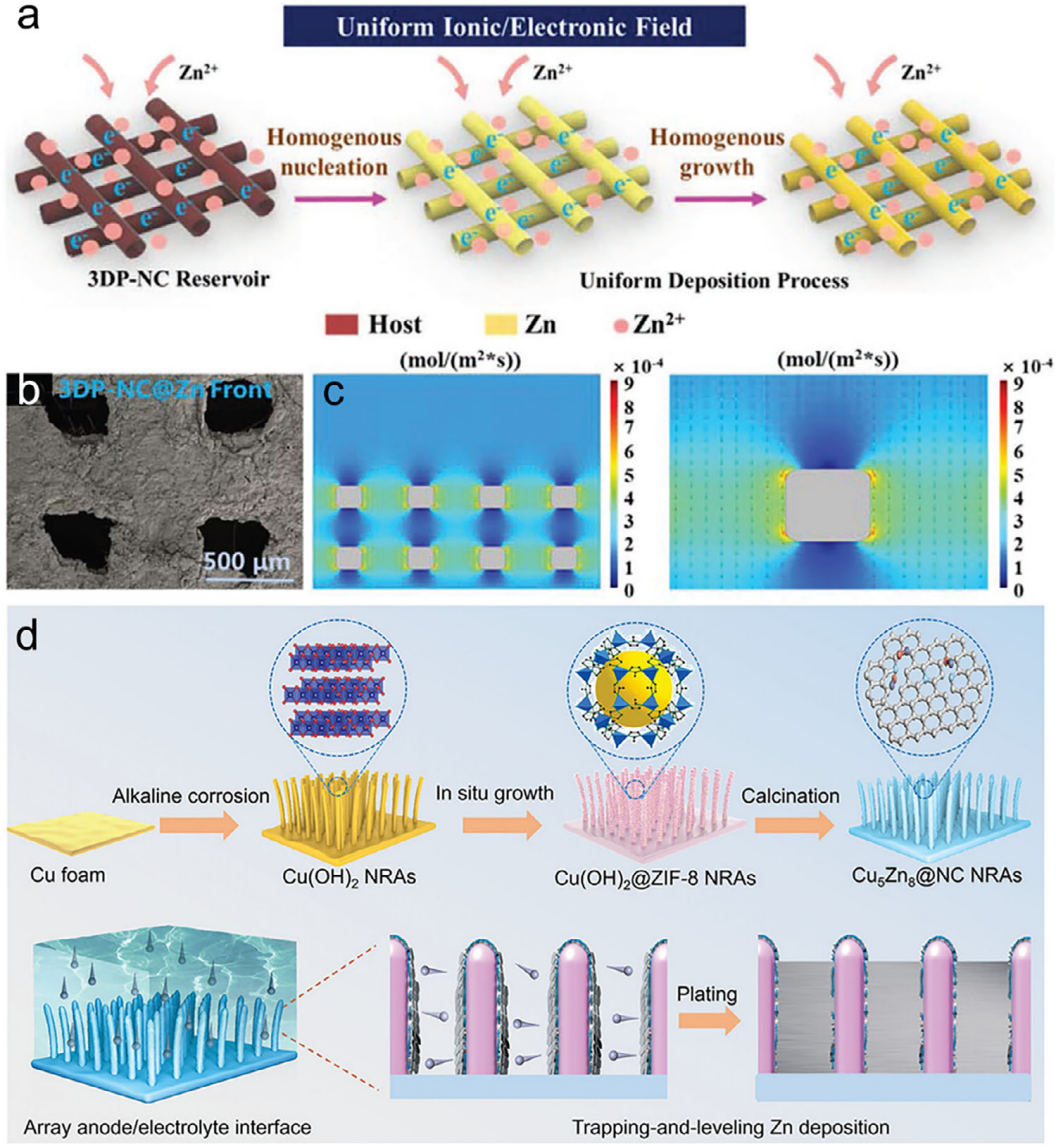
(a) Schematic illustration of the underlying dendrite growth mechanism for bulk‐NC@Zn and 3DP‐NC@Zn electrodes. (b) SEM images of the 3DP‐NC@Zn electrodes after cycling. (c) Ionic flux distribution of 3DP‐NC hosts simulated by COMSOL Multiphysics [74]. Copyright 2022, Wiley‐VCH. (d) Schematic illustration for the synthesis of the Cu_5_Zn_8_@NC NRAs and the schematic diagram of the Zn deposition mechanism [50]. Copyright 2024, Wiley‐VCH.

Nowadays, advanced conductive hosts with various 3D architectures show their essential superiorities in restraining Zn dendrite growth by optimizing Zn^2+^ ion flux, homogenizing the distribution of the interfacial electric field, and decreasing the local current density [78, 178]. For instance, Li et al. [74] have prepared 3D N‐doped carbon (3DP‐NC) host with regular square holes by the 3D printing technique and the follow‐up heat‐treatment method. As shown in Figure 9a, 3DP‐NC hosts with a large zincophilic surface efficiently homogenize the distribution of the interfacial electric field and reduce the local current density. Besides, the highly ordered 3DP‐NC with micron‐sized channels optimizes interfacial Zn^2+^ ion flux, which achieves rapid ion transport kinetics and quasi‐steady‐state ionic supplements during Zn deposition. The collective benefits of 3DP‐NC effectively relieved concentration polarization and endowed the 3DP‐NC@Zn anode with dendrite‐free morphology. Oppositely, the Bulk‐NC host with low surface area drove inhomogeneous Zn nucleation and growth, which induced uneven distribution of electric/ionic field. Of note, the uncontrollable ion diffusion motivated by the uneven electric field caused unrestricted 2D diffusion of Zn^2+^ ions and aggravated the concentration polarization, further promoting the Zn dendrite growth [179]. Furthermore, the 3DP‐NC@Zn anode showed a relatively smooth morphology after cycling compared with the Bulk‐NC@Zn (Figure 9b). Clearly, the scanning electron microscopy (SEM) images of cross profile illustrated horizontal growth of Zn on the interior surface. On the contrary, the randomly distributed bulk‐like Zn dendrites covered the whole surface of the Bulk‐NC after cycling. The structural superiority of the 3DP‐NC host compared with the Bulk‐NC was testified via the simulation of interfacial Zn^2+^ ion flux after Zn deposition. The privileged structure of the 3DP‐NC host achieved the redistribution of the interfacial ionic field and ensured higher Zn^2+^ ion concentration within the multi‐channel structure rather than on the top surface (Figure 9c). The maximum ionic flux on the walls of the reservoirs was more beneficial to induce preferential nucleation along the internal surface and trigger the Zn deposition into a horizontal growth pattern. In contrast, the generous Zn^2+^ ions were concentrated around the tips of Bulk‐NC and exacerbated the concentration polarization, thereby promoting rapid Zn dendrite growth. As a result, the uniform Zn deposits covered the whole surface of the 3DP‐NC host without any dendrites. The optimized 3DP‐NC host delivered a low overpotential and a high CE, endowing the 3DP‐NC@Zn composite anode with the dendrite‐free morphology and long cycling lifespan in the symmetrical cell. Recently, Tian et al. [50] developed Cu_5_Zn_8_ nanorod arrays coated with N‐doped carbon layers on Cu foam as the 3D zincophilic host (Cu_5_Zn_8_@NC NRAs) via a multi‐step etching strategy (Figure 9d). The COMSOL simulations revealed that the Cu_5_Zn_8_@NC NRAs featuring aligned ion channels, sufficient inner volume, and a large specific surface reduced the local current density, homogenized the electric field distribution and zinc ion flux, and mitigated concentration polarization at the electrolyte‐electrode interface. As a result, the Cu_5_Zn_8_@NC NRAs provided highly ordered Zn^2+^ ion tunnels to optimize ion diffusion and thus achieve a confined Zn plating within the interspace of nanorod arrays, ensuring dendrite‐free morphology during a prolonged plating/stripping process.

Similarly, Lu's group has employed the 3D carbon nanotube (CNT) frameworks on carbon cloth with high specific surface area as the host to achieve the high‐performance ZMAs [180]. Simulation results of electric field distribution verify that 3D CNT hosts effectively lower local current density and homogenize the interfacial electric field, which facilitates homogeneous Zn deposition. The dendrite‐free Zn/CNT composite electrode experienced a prolonged cycle life of 200 h with significantly reduced voltage hysteresis. Besides, the Zn/CNT||MnO_2_ battery also delivered a substantial lifespan over 1000 cycles with 88.7% capacity retention. Moreover, Zhang et al. [181] have in situ integrated the CuO nanowires on the Cu mesh as the multi‐structural hosts to achieve the redistribution of the electric field. The interlaced 3D Cu@CuO skeletons featuring a homogeneous interfacial electric field and a decreased local current density achieved a continuously uniform Zn deposition, thus endowing the composite ZMAs with low voltage hysteresis and long‐term cycling stability.

#### 3.2.2 Constructing Functional Interlayer

**FIGURE 10:**
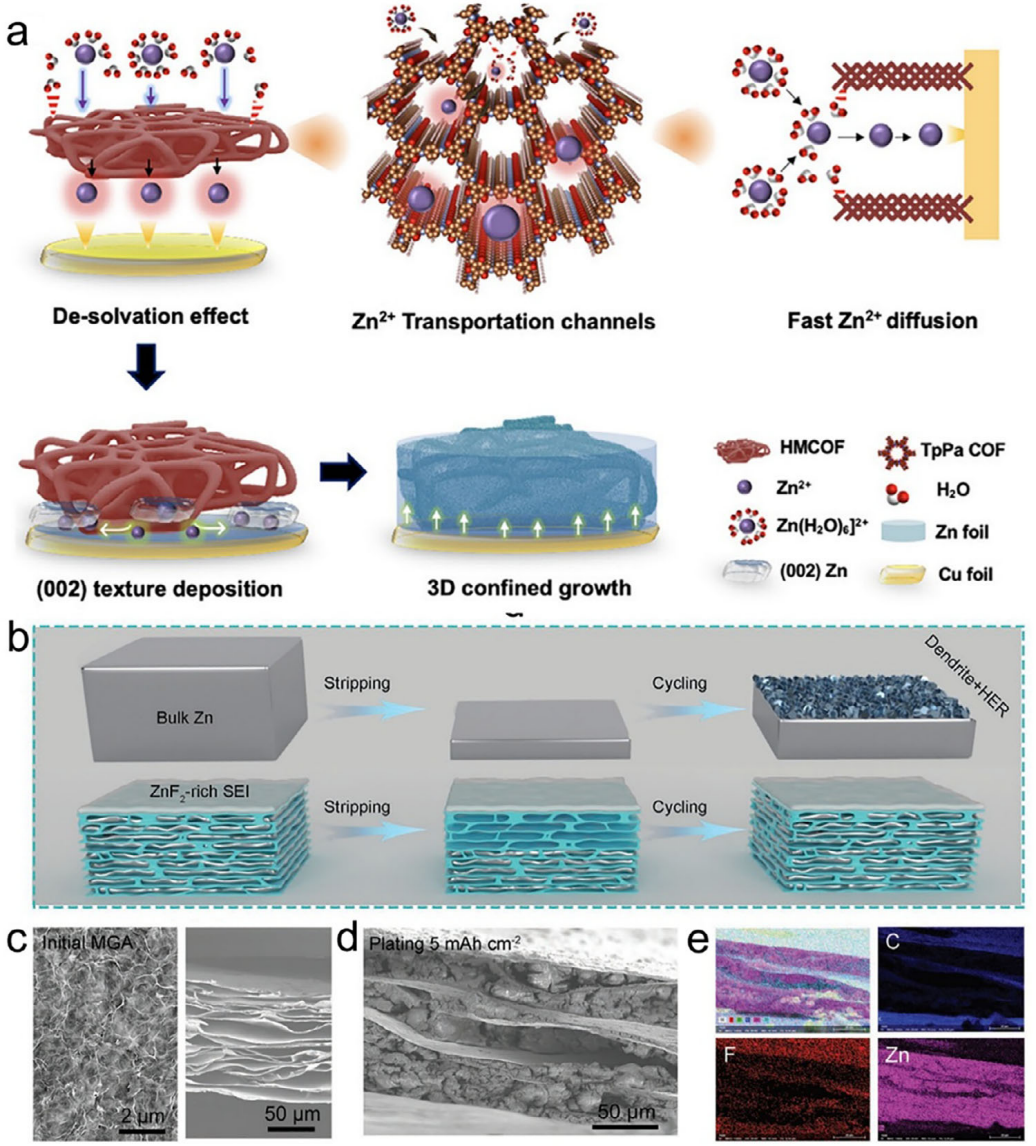
(a) Schematic illustration of Zn deposition on HMCOF‐Cu [103]. Copyright 2025, Wiley‐VCH. (b) Schematic illustration of Zn plating and cycling on bulk Zn foil and MGA@Zn electrodes (lower). (c) SEM images of top (left) and side views under unconstrained conditions for the MGA material, (d) Side‐view image and (e) corresponding elemental mapping of the MGA sample after plating 5 mAh cm^−2^ [182]. Copyright 2022, Wiley‐VCH.

Coating a functional interlayer with an ion transport channel as another pivotal strategy both accelerates desolvation of hydrated Zn^2+^ ions at the host‐electrolyte interface and suppresses water‐related side reactions by isolating electrolytes [63, 110]. Recently, Yu et al. [103] developed a gradient 3D host via integrating hollow microfibers of covalent organic frameworks on Cu foil (HMCOF‐Cu) for achieving stable bottom‐up Zn plating. The top HMCOF layer featuring numerous electronegative carbonyl (C = O) groups strongly interacts with the active H_2_O in the first solvation shell, thereby facilitating a rapid desolvation of hydrated Zn^2+^ (Figure 10a). Leveraging the high electrical conductivity and superior zincophilicity, the Cu foil at the bottom provided favorable nucleation sites to induce a preferential Zn nucleation. What's more, the 3D HMCOF‐Cu frameworks with hierarchical pores and sufficient internal space accelerated the Zn^2+^ ion diffusion kinetics via the boosted capillary effect and accommodated bottom‐up, gradient Zn deposition. Expectedly, the 3D HMCOF‐Cu host reinforced the stability of the Zn plating/stripping process and enabled the ZMAs with a long‐cycle life over 2500 h at 10 mA cm^−2^. Furthermore, Zhou et al. [182] designed the 3D MXene/graphene aerogel (MGA) frameworks by the oriented freezing process as the zincophilic hosts for constructing high‐performance ZMAs. Compared with bare Zn, the 3D MXene/graphene hosts could induce an in situ formed ZnF_2_‐rich SEI at the interface between the composite anode and electrolyte, thus suppressing the parasitic reactions and achieving preferential removal of Zn^2+^ solvation sheath (Figure 10b). The top and cross SEM images revealed that MGA provided a flat top surface with no visible pores and abundant space inside the hosts (Figure 10c). Benefiting from the desirable architecture, the metallic Zn was successfully encapsulated within the interior of the MGA structure after Zn deposition (Figure 10d). Besides, the corresponding element mapping also proved a homogeneous distribution of metallic Zn within the hosts (Figure 10e). Remarkably, the evolved ZnF_2_‐rich interface layer during cycling on the top surface of MGA could allow the diffusion of Zn^2+^ ions and decrease the contact area between electrolyte/electrode, thereby limiting parasitic reactions. The in situ optical microscopy visualization results revealed numerous H_2_ bubbles generated on the backside of Cu foil after aging for 12 h. Moreover, loose and irregular Zn dendrites grew on the Cu foil after deposition. On the contrary, there were no bubbles and Zn dendrites on the MGA host regardless of the aging or Zn deposition process. According to differential electrochemical mass spectrometry (DEMS) profiles, the H_2_ evolution rate of the MGA@Zn electrode was much lower than that of Cu@Zn, which further demonstrated the inertness of MGA for HER. Meanwhile, no irreversible production of Zn_4_(SO_4_)_4_(OH)_6_·5H_2_O was observed during the charging/discharging process, which was certified by the in situ XRD and Raman characterizations. As expected, the MGA host exhibited a relatively smooth and uniform upper surface after one cycle. Meanwhile, the MGA host showed a superior CE at a high current density compared with Cu foil, endowing the corresponding symmetric battery using the MGA@Zn electrode with long cycling life. Besides, Chen et al. [183] reported stable composite ZMAs with an inert ZnO layer, which was designed using the 3D Cu skeleton by a thermal infusion strategy. During the melting‐wetting‐cooling process of metallic Zn, the inert ZnO layer formed on the metallic Zn surface, which could efficiently inhibit the formation of Zn dendrites and side reactions by isolating the electrolyte. Owing to the superior reversibility, the composite ZMAs revealed a long lifespan with low voltage hysteresis. Meanwhile, the corresponding full batteries showed high‐rate performance and capacity retention after cycling.

### 3.3 Spatial Confinement

Although commercial 3D conductive hosts with increased surface area have made considerable progress in suppressing dendrite growth, the structural variations in hundreds of micrometers would cause an ultra‐long length of electrons and ions [184, 185]. The ultra‐long length of electrons with hundreds of micrometers of 3D conductive hosts and the sluggish ion diffusion rate motivate the Zn deposition into a “top growth” mode during the initial Zn deposition process, which cannot completely mitigate the dendrite formation [59, 99]. Moreover, the disordered structure of 3D conductive hosts would hinder the electrolyte from fully contacting the whole frameworks, thus causing uneven distribution of Zn^2+^ ion flux. Based on this consideration, realizing uniform Zn deposition within the 3D conductive hosts has been one of the research hot spots, so‐called spatial confinement effect [179]. As mentioned above, the 3D conductive hosts with desired structural features could effectively decrease local current density and uniform the electric field, achieving precise manipulation of Zn^2+^ ion flux from the bulk electrolyte to the electrode interior. Moreover, the 3D hosts can effectively immobilize Zn^2+^ ions within the space of frameworks by constructing a hierarchically layered structure with tunable pore sizes and composition, thus achieving sequential deposition from the bottom to top or inside to outside [45, 48, 59, 101]. Furthermore, the 3D porous hosts with sufficient internal space effectively utilize spatial confinement effects and alleviate structural stress caused by volume expansion, thus making it go on forever until the Zn deposits fill the hosts [98].

#### 3.3.1 Designing Ordered Porous Structure

**FIGURE 11:**
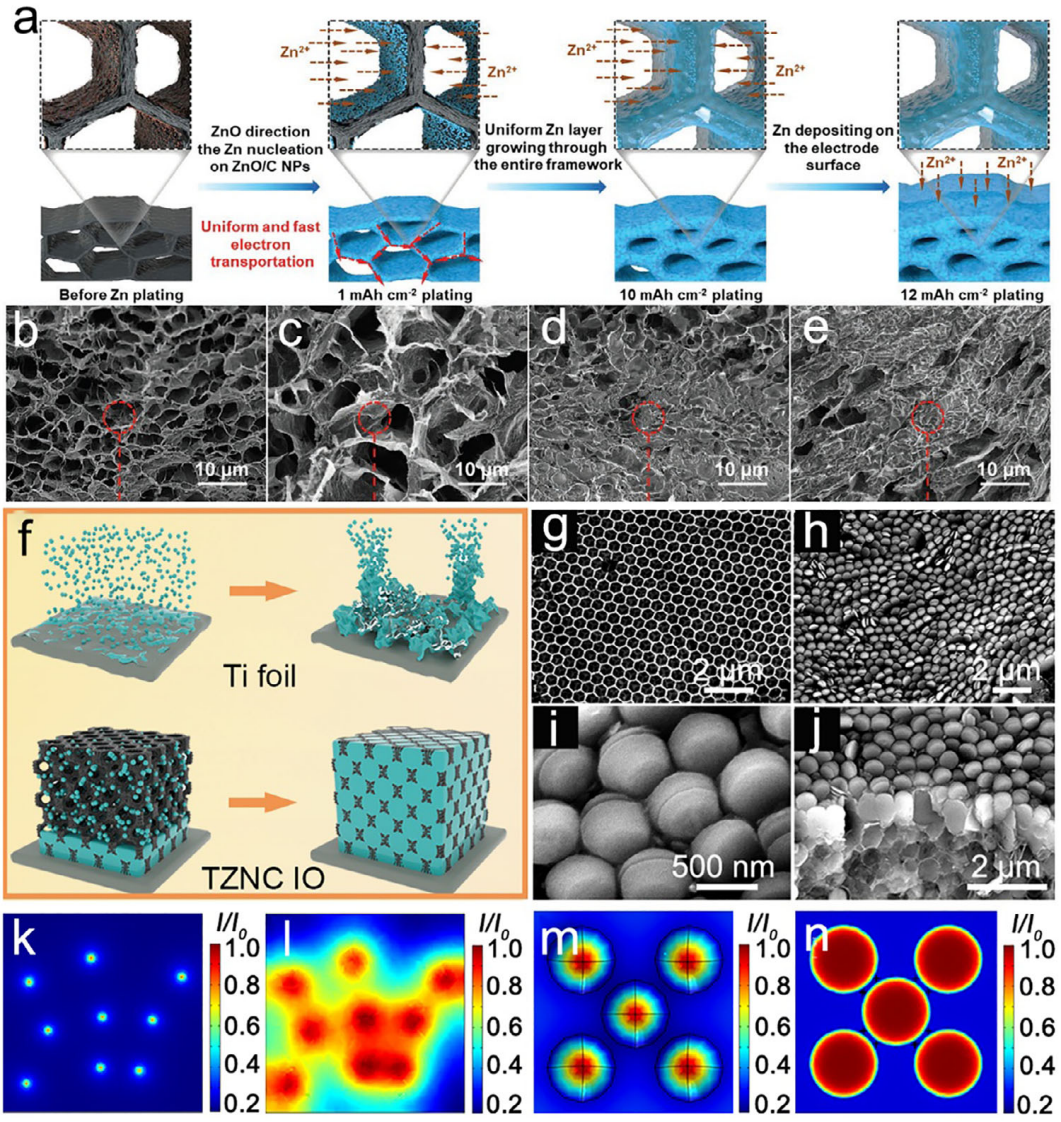
(a) Schematic illustration of Zn deposition principle on the 3D‐ZGC host. SEM images of the 3D‐ZGC scaffold with different Zn deposition capacities for (b) 1 mAh cm^−2^, (c) 5 mAh cm^−2^, (d) 10 mAh cm^−2^ and (e) 12 mAh cm^−2^ [80]. Copyright 2022, Wiley‐VCH. (f) Schematic illustration of Zn deposition on different hosts. SEM images of the (g) TZNC host and (h‐j) TZNC@Zn. Simulation results of the current density distribution on the surface of (k) Ti foil, (l) Ti@Zn, (m) TZNC host, and (n) TZNC@Zn [78]. Copyright 2022, Wiley‐VCH.

Designing conductive hollow 3D hosts with porous structure has been regarded as a favorable method for achieving spatial confinement deposition. For instance, Xue et al. [80] developed ZnO/C nanoparticles decorated 3D porous graphene‐carbon nanotubes scaffolds (3D GO‐CNTs) as a zincophilic host via the nanocomposite gelation technology and the subsequent ice‐templated freeze‐drying method for constructing high‐performance ZMAs. The fundamental principle of Zn deposition on a 3D‐ZGC host with different capacities was revealed in Figure 11a. The conductive skeleton with a width of ten microns was more favorable for electrolyte penetration and uniform electron transfer, thereby leading to homogeneous Zn^2+^ ion flux and rapid ion diffusion within the host. The simulation results of the 3D‐ZGC host in the aspect of local current density and electric field distribution further revealed the structural effects for regulating Zn deposition behavior. The interconnected porous framework with large surface area could efficiently reduce the current density and ensure uniform charge transfer. Furthermore, the achieved homogeneous distribution of electric field originating from the redistributed charges was beneficial to achieving a uniform Zn^2+^ ion flux. On the contrary, the current density near the isolated protuberance on the surface of bare Zn foils was much higher than that of other regions, resulting in a significantly uneven electric field distribution. Moreover, the ZnO/C nanoparticles as zincophilic sites induced a preferred deposition in the framework and then led to the Zn deposits covering the whole surface of the 3D‐ZGC porous host. The experimental results proved that the 3D‐ZGC host showed a negligible morphology transition at the early stages of the deposition (Figure 11b). With the increased deposition capacity, the Zn deposits continuously grew and filled the space between ZnO/C NPs, ultimately covering the internal surface of the framework and forming a smooth deposition layer (Figure 11c,d). In the subsequent deposition process, most of the holes were filled with metallic Zn and then induced the Zn deposits to coat the whole surface (Figure 11e). As a result, the 3D‐ZGC host achieved a spatial confinement deposition with the orientation from the bottom to the top. The 3D‐ZGC host has synergistically utilized the superiorities of both 3D porous structure and zincophilic species, thereby effectively hindering the Zn dendrite growth by the spatial confinement effect. As expected, the 3D‐ZGC host exhibited significantly reduced overpotential and high CE, which endows the Zn@3D‐ZGC electrode with a long‐term cycling lifespan over 1000 cycles and low voltage hysteresis. Although the spatial confinement effect was achieved by the 3D‐ZGC host, the top deposition would not be completely eliminated owing to the irregular spatial structure. The out‐of‐order spatial architectures of 3D porous hosts such as the randomly scattered channels and different‐aperture holes generally hinder the Zn^2+^ ion diffusion within the frameworks, thereby resulting in the heterogeneous distribution of Zn^2+^ ion flux. Beyond that, the top surface with high curvature is another crucial reason for the formative top deposition because of the “tip effects”. These structural drawbacks will deteriorate ion diffusion kinetics and form the void space within the hosts, thus reducing the energy density of the full battery.

With an in‐depth study of the mechanism for Zn deposition, a great deal of research has proved that highly ordered architectures with aligned channels and pores are competent for optimizing the ion diffusion kinetics owing to their organized charge transport pathways and uniform electric field distribution. Sun et al. [78] synthesized a 3D hollow conductive host with a highly ordered inverse opal structure and the zincophilic TiO*_x_*/Zn/N‐doped carbon (denoted as TZNC IO) through a facile hard template strategy. The ordered inverse opal structure achieved a precise spatial confinement effect, where the metallic Zn filled the frameworks without any void space after deposition. The advanced synthetic method of TZNC IO was developed using the polystyrene (PS) colloidal crystal template and subsequent in situ topology transformation technology. Firstly, the PS spheres with uniform particle size were integrated on the Ti foil to spontaneously form the close‐packed opal array by an evaporation‐induced self‐assembly strategy. Then, the prepared opal array was sequentially infiltrated with the precursor solution and annealed at the ideal temperature to obtain TiO*_x_*/ZnO IO. With the goal of increasing the zincophilicity, the TiO*_x_*/ZnO IO was in situ converted to TiO*_x_*/zeolitic imidazolate framework (ZIF) IO by long‐term immersion in 2‐methylimidazole solution. Finally, the zincophilic TZNC IO was fabricated via annealing TiO*_x_*/ZIF IO under N_2_ at the optimal temperature. The stepwise synthetic method realized precise synergism with structural modulation and composition regulation, which plays a crucial role in achieving high‐performance ZMAs. The morphology evolution of Zn deposits on different hosts was illustrated in Figure 11f. Clearly, randomly distributed Zn flakes with different lengths covered the surface of Ti foil, eventually forming irregular Zn dendrites. In contrast, metallic Zn filled the TZNC IO host without Zn dendrites after the deposition process. The SEM images of the TZNC IO host before and after deposition illustrated that the metallic Zn deposited within the void space of the TZNC IO, thereby growing into the spherical particles owing to the spatial confinement effect (Figure 11g–j). Furthermore, the corresponding cross‐sectional image revealed that the compact Zn deposits were connected to the TZNC IO host without the void space, further proving precisely controllable Zn growth. Simulations of current density distribution of 2D Ti foil and 3D TZNC IO host were applied to investigate the principle of Zn growth during the deposition process. The local current density on unevenly distributed tips of Ti foil was much higher than in the flat region, which would cause charge polarization and a heterogeneous Zn^2+^ ion flux, thereby leading to Zn dendrite growth. Besides, the aforesaid inhomogeneity enlarged with the Zn deposition owing to the Zn dendrite growth, further accelerating the dendrite growth rate (Figure 11k,l). On the contrary, the TZNC host with a highly ordered structure and large specific surface area effectively decreased the current density and homogenized the charge distribution, thus forming a uniform electric field on the top surface (Figure 11m). The periodically arranged distribution of current density was achieved by the honeycombed TZNC IO, where the charge center is always within the pores, thus enabling the Zn^2+^ ions enriched in the bottom and leading to preferred nucleation and growth (Figure 11n). The improved zincophilicity originating from the amorphous TiO*_x_* and Zn/N‐doped carbon was an important factor for realizing the spatial confinement deposition. Owing to the aforementioned superiorities, the TZNC host showed low nucleation overpotentials and high CE of 98.6%, thus endowing the TZNC@Zn electrode with a long lifespan over 450 h and low voltage hysteresis. In addition, Duan et al. [75] designed the desirable 3D Ni hosts with ordered architecture through combining the 3D printing technology and subsequent electrodeposition method. The highly ordered 3D Ni hosts could achieve a redistribution of the electric field, further inducing the preferential Zn deposition inside the skeletons without dendrite growth. Moreover, the smooth surface with low height difference was maintained on the cycled 3D Ni‐Zn electrode, which proved the 3D ordered structure could effectively hinder dendrite growth. Benefiting from the structural advantages, the 3D Ni‐Zn electrode showed a high reversibility with satisfactory CE, endowing the corresponding full cell with superior long‐term stability. Analogously, a 3D Ti host with a continuous porous structure designed by Qian's group could also ensure a homogeneous electric field and mitigate the volume expansion, thereby suppressing the top deposition and dendrite growth [68]. Benefiting from the structural advantages, the Ti/Zn anode delivered a high cycling stability of more than 2000 h in symmetrical cells.

#### 3.3.2 Designing Hierarchical Structure with Adjustable Pore Sizes

**FIGURE 12:**
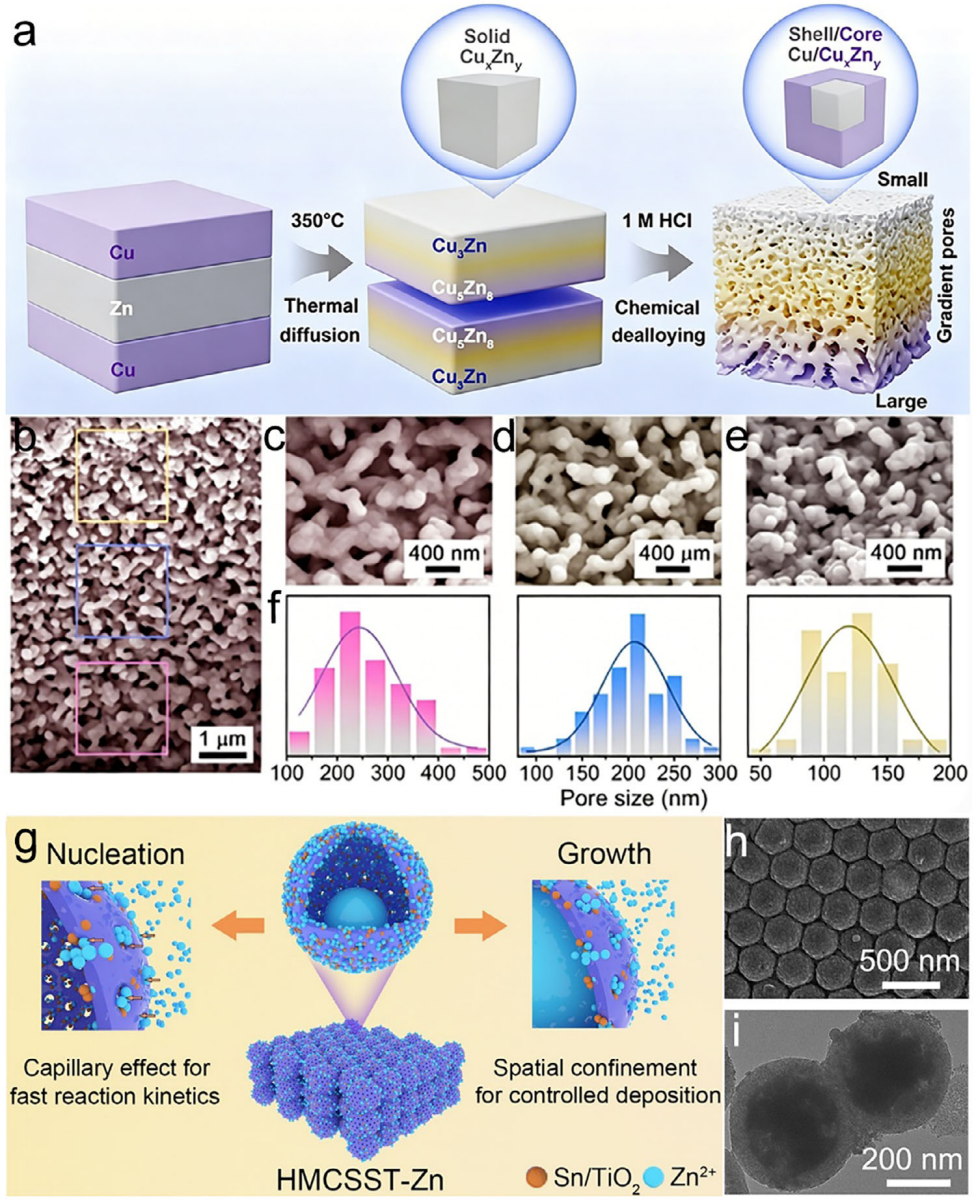
(a) Schematic diagram for the synthesis of a GNP Cu‐Zn alloy electrode. (b) Representative SEM image of the cross‐sectional GNP Cu/Cu*_x_* Zn*_y_* electrode. Typical SEM images at the bottom (c), middle (d), and top regions (e) of the GNP Cu/Cu*_x_* Zn*_y_* electrode. (f) The corresponding nanopore size distributions [52]. Copyright 2025, American Chemical Society. (g) Schematic illustration of Zn deposition on HMCSST hosts. (h) SEM images of the HMCSST host. (i) TEM images of HMCSST‐Zn anode [98]. Copyright 2024, Wiley‐VCH.

Hierarchical structures with gradient pores and compositions enable precise physical confinement of Zn deposition by optimizing ion transport and reaction kinetics. For instance, Liu et al. [52] fabricated a 3D Cu/Cu*_x_* Zn*_y_* network with pore and composition gradients via a facile alloying/dealloying strategy as a zincophilic host (GNP Cu/Cu*_x_* Zn*_y_*) to achieve spatially confined Zn deposition (Figure 12a). Cross‐sectional SEM images indicated that GNP Cu/Cu*_x_* Zn*_y_* possessed a hierarchical structure composed of interconnected gradient nanopore channels, where pore sizes increased progressively from top to bottom (Figure 12b–f). This gradient nanoporous structure within GNP Cu/Cu*_x_* Zn*_y_* stemmed from the partially selective etching of Zn metal in the gradient Cu*_x_* Zn*_y_* alloy. The well‐designed structure with gradient nanopore channels guaranteed the efficient utilization of ion‐diffusion and electron‐transport pathways during the Zn plating/stripping process. Meanwhile, gradient nanopore channels modulated the solvation shell of hydrated Zn^2+^ ions within the electrical double layer, enabling accelerated desolvation kinetics. Additionally, the calculations indicated that the Cu_5_Zn_8_ alloy as the zincophilic core at the bottom of the framework offered high adsorption energy for Zn atoms and reduced the Δ*G*_nucleation_, ultimately guiding a preferential Zn nucleation within the host. Benefiting from the aforementioned merits, the GNP Cu/Cu*_x_* Zn*_y_* host suppressed Zn dendrite growth and enabled a dendrite‐free, highly reversible Zn plating/stripping process, achieving an average CE of 99.3%. Moreover, the Cu/Cu*_x_* Zn*_y_* electrode exhibited superior cycling durability for more than 6700 h at 0.5 mA cm^−2^ and excellent rate capability, ensuring the GNP Cu/Cu*_x_* Zn*_y_* ||Zn*_x_* V_2_O_5_/C full cells operated stably for over 5000 cycles at 5 A g^−1^. Furthermore, Yu et al. [98] designed a hollow mesoporous carbon sphere opal array (HMCSST) with well‐dispersed Sn/TiO_2_ nanoparticles via successful template removal and phase evolution as a favorable zincophilic host for highly reversible ZMAs. Leveraging the robust capillary effect endowed by the ordered hierarchical porous architecture, Zn^2+^ ions accumulated preferentially inside the hollow mesoporous carbon spheres and established a distinct inward‐to‐outward concentration gradient, consequently accelerating Zn^2+^ diffusion kinetics and triggering a preferential Zn nucleation within the HMCSST hosts (Figure 12g). The single hollow‐mesoporous sphere within the HMCSST functions as a well‐defined nanoreactor that spatially confine the deposition reaction, ensuring a spatiotemporally controlled growth pattern (Figure 12h, i). Leveraging these structural advantages, the HMCSST‐Zn symmetric cell exhibited superior electrochemical kinetics characterized by remarkably reduced activation energy (21.1 kJ mol^−1^) for the Zn deposition reaction. Moreover, the incorporation of Sn/TiO_2_ clusters within the host could both improve the zincophilicity and suppress the HER, leading to a lowered overpotential for Zn deposition and reinforcing the thermodynamic stability of ZMAs with a high average CE of 99.7% over 300 cycles. Furthermore, the HMCSST‐Zn electrode sustained an extended lifespan of over 1300 h at 37.5% depth‐of‐discharge (DOD) in a symmetric cell, and thus achieved stable operation for more than 5000 cycles in HMCSST‐Zn||NVO full cell.

Besides, Shen et al. [184] constructed porous hosts with a stratified deposition framework (SDF) that features various nucleation overpotentials, which could achieve orderly deposition from the bottom to top. Concretely speaking, the NiO layer with high overpotential on the top surface provided weaker interaction with metallic Zn, thus avoiding the top deposition on the electrode/electrolyte interface. Besides, the super‐hydrophilic characteristics of SDF enabled the great permeability of Zn^2+^ ions within the hosts. Meanwhile, the copper foam and nickel foam with lower overpotential on the bottom could provide stronger binding energy for metallic Zn and induce preferential Zn deposition, thus achieving the deposition model of “bottom to top”. Beyond that, a novel 3D conductive host composed of interconnected hollow carbon fibers with even‐distributed TiO_2_ and SiO_2_ (HSTF) has been developed for achieving high‐performance composite ZMAs [77]. The morphology evolution of Zn deposits on the 3D HSTF with different deposition capacities proved that the hollow fibers could effectively absorb Zn^2+^ ions within the space of frameworks, achieving a sequential deposition from interior to exterior. The DFT calculation results revealed that the amorphous SiO_2_ and TiO_2_ could provide strong binding energy with metallic Zn, which was consistent with the results of preferential deposition, thus avoiding the top deposition. Besides, the results of COMSOL simulation proved that the 3D hollow HSTF host could reduce the local current density and homogenize the electric field distribution owing to the increased specific surface area. Benefiting from these superiorities, the 3D hollow HSTF with multilevel channels could effectively uniform the Zn^2+^ ions flux and realize the spatial confinement deposition within the host, thereby achieving a smooth and compact Zn deposition morphology. As a result, the 3D‐HSTF hosts showed the low overpotential and high average CE value, endowing the composite anode with significantly enhanced lifespan. Moreover, a eutectic alloying composed of alternating Zn and Al nanolamellas (Zn_88_Al_12_) was also proposed to achieve spatially confined deposition owing to their lamellar structure [8]. By virtue of symbiotic less‐noble Al lamellas, the in situ formed insulating Al_2_O_3_ shell on the top could not only hinder the formation of irreversible by‐products but also guide subsequent growth of Zn.

#### 3.3.3 Alleviating Structural Stress

**FIGURE 13:**
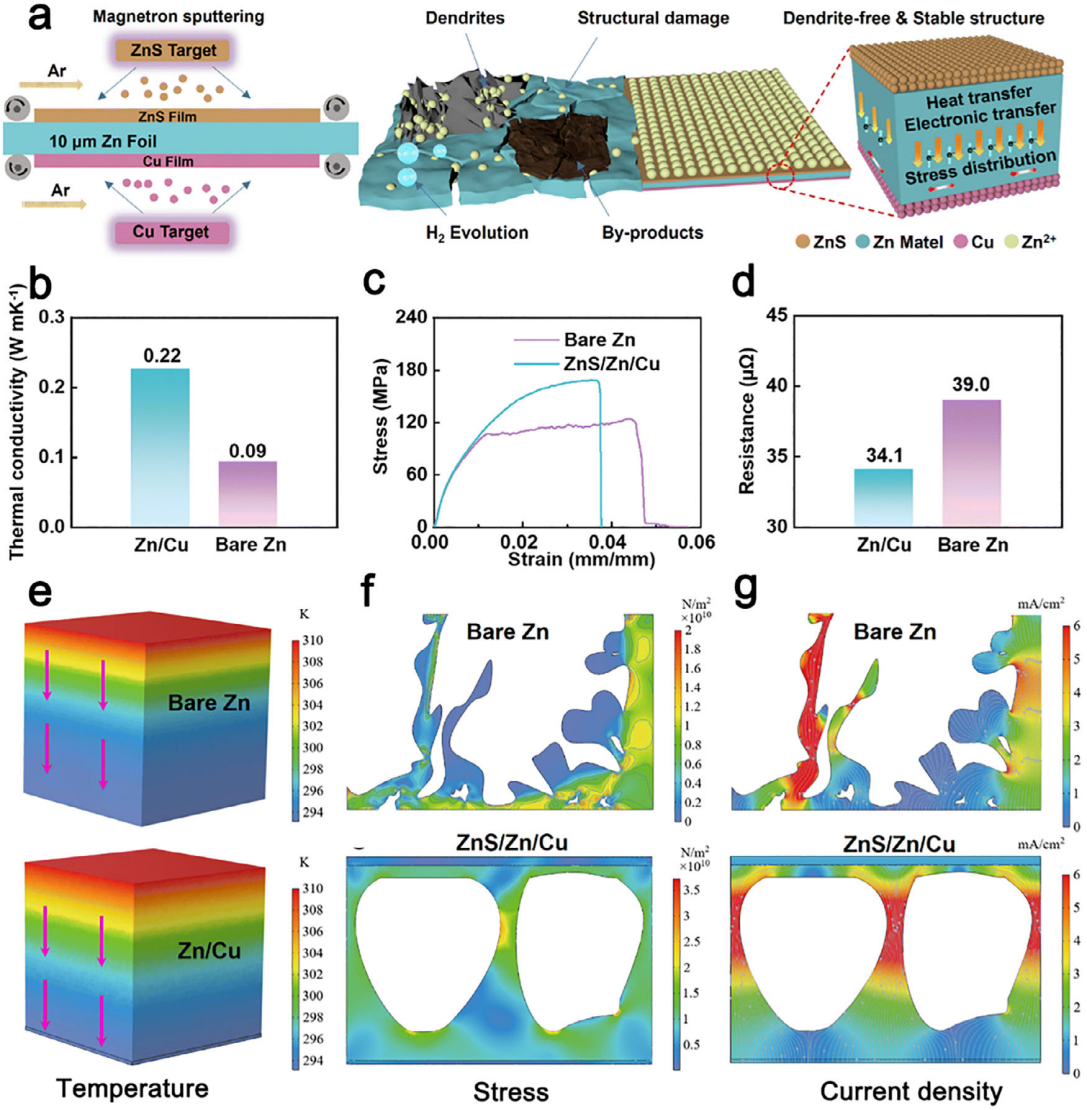
(a) Schematic diagram for the fabrication of ZnS/Zn/Cu electrode. (b) Thermal conductivity, (c) stress curve, and (d) resistance value of different electrodes. (e) Temperature distribution, (f) stress distribution, and (g) current density of different electrodes [48]. Copyright 2024, Royal Society of Chemistry.

The volume variation of ZMAs during the Zn plating/stripping process inevitably induces residual stress within the electrode. Specifically, Zn metal with high Young's modulus and poor plastic deformability enables a dramatic volume fluctuation during Zn deposition, which consequently gives rise to the accumulation of internal residual stress. The structural stress continuously accumulates at nucleation sites, thus triggering uneven Zn deposition and deteriorating interfacial reaction kinetics. Ultimately, the accumulation of uneven structural stress during Zn deposition induces the generation of microcracks and drives preferential zinc growth along crack edges, ultimately causing severe structural collapse. This problem will be exacerbated under high current densities and deep discharge states. Nowadays, effectively alleviating the mechanical stress generated during Zn electrodeposition is crucial for fabricating dendrite‐free ZMAs. Space‐confined strategies can mitigate structural stress induced by volume expansion during Zn deposition and inhibit irreversible structural failure. For instance, Guan et al. [186] proposed a double‐sided engineering strategy for preparing stable ZMAs with a ZnS functional interfacial layer on the top and a conductive Cu host at the bottom, which achieves spatially confined and highly reversible Zn plating/stripping (Figure 13a). Such a unique structure enables synergistic optimization of the thermal field, stress field, and electric field. The copper host with favorable thermal conductivity accelerates internal‐to‐external heat transfer and restricts dendrite growth induced by localized high temperature (Figure 13b). Simulation results revealed that the dramatic volume fluctuation and uneven stress distribution of host‐free ZMAs during plating/stripping generated abundant cracks and voids and caused irreversible structural deterioration (Figure 13c). Meanwhile, generated cracks and voids block electron transport pathways, leading to high charge transfer resistance and inhomogeneous electric field distribution (Figure 13d). Conversely, the Cu host relieves stress concentration during Zn plating/stripping, endowing the ZnS/Zn/Cu electrode with rapid heat transfer, uniform stress distribution, and homogeneous electric field distribution (Figure 13e–g). Additionally, the ZnS functional interfacial layer inhibits parasitic reactions, restricts top deposition, and optimizes Zn^2+^ ion flux, which is favorable to homogeneous Zn deposition. Thanks to these superior properties, the ZnS/Zn/Cu electrode realized space‐confined reversible Zn deposition and delivered stable cycling performance over 300 h under a high DOD of 85.5%. Recently, Yu et al. [57] developed an effective assembly strategy to synthesize Ag‐modified reduced graphene oxide foam (denoted as AGF) with interconnected and dense pore channels as a Zn host. The robust Ag‐rGO channels and the strong synergistic effect between rGO and Ag collectively endow the AGF host with exceptional superelasticity and mechanical stability, readily accommodating drastic pressure fluctuations under extreme conditions. The superelastic and robust AGF host with sufficient internal space can relieve the structural stress of Zn deposition, enabling the AGF@Zn electrode to cycle stably for over 120 h under a stack pressure of 26 MPa. Moreover, flexible and scalable graphene nanosheets can bear the strain of ZMAs during cycling and mitigate volume expansion. The graphene‐based 3D host exhibits remarkable flexibility and retains structural stability under bending deformation, which renders it applicable for highly stable ZMAs. For instance, Cao et al. [105] fabricated vertically aligned N‐doped graphene (N‐VG) nanosheet arrays on carbon cloth (N‐VG@CC) as an advanced 3D host, thereby constructing a 3D Zn@N‐VG@CC anode with both good electrochemical performance and mechanical flexibility. Moreover, Zeng et al. [19] synthesized nitrogen‐doped graphene nanofiber clusters (GFs) and vertical graphene arrays (VGs) modified multichannel carbon matrix (3D‐FGC) as a 3D host (3D‐RFGC) via thermal‐chemical vapor deposition (T‐CVD) strategy, which realizes the confined growth of Zn metal along the interconnected 3D channels of vertical graphene. The flexible graphene nanofiber clusters effectively release structural stress and offer abundant void space for Zn deposition, which mitigates volume variation and boosts the utilization of ZMAs. Consequently, the 3D‐RFGC host greatly improved the cycling stability and service life of the ZMAs. The symmetric 3D‐RFGC@Zn||3D‐RFGC@Zn cells exhibited stable plating/stripping cycles for up to 7300 h at a current density of 1 mA cm^−2^, far exceeding bare Zn foil and Zn foam.

## 4 Conclusions and Outlook

**FIGURE 14:**
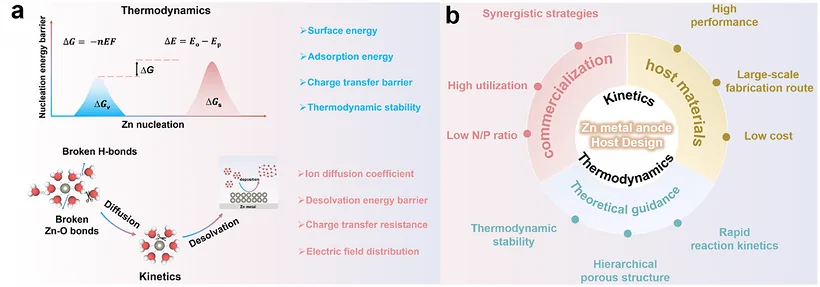
(a) Thermodynamic and kinetic mechanisms of Zn deposition. (b) The perspectives and suggestions for advanced host designs.

AZMBs have been regarded as a promising alternative for next‐generation safe batteries because of the superiorities in high theoretical capacity, abundant resources, and intrinsic safety. The conductive hosts as a pivotal component for building the complete ZMAs have direct effects on the aspect of morphology evolution during Zn deposition, which is closely related to the electrochemical performance. The rational design of 3D conductive hosts is favorable for achieving a stable ZMA without dendrites owing to the potential advantages in synergism of thermodynamic and kinetic manipulation. In this review, the recent advances of 3D conductive hosts and their design principles in terms of structural modulation and composition regulation are summarized, especially highlighting how the hosts manipulate the thermodynamic and kinetic factors during Zn deposition. The surface energy, adsorption energy, charge transfer barrier, and intrinsic thermodynamic stability of conductive substrates can be precisely modulated through surface modification and interfacial engineering strategies, which effectively decrease Zn nucleation overpotential and increase the energy barrier of HER, facilitating homogeneous Zn nucleation and suppressing adverse parasitic reactions (Figure 14a). Moreover, rational structure design of 3D hosts can homogenize interfacial electric field distribution and reduce local current density, which improves the ion diffusion coefficient, accelerates the ion desolvation process, and reduces charge transfer resistance, thus optimizing electrochemical reaction kinetics and restraining Zn dendrite growth. The advanced design strategies of 3D conductive hosts for high‐performance ZMAs summarized in this review are presented in Tables 1, 2, 3. In spite of great achievements in high‐performance 3D conductive hosts, further developing stable ZMAs featuring high energy density, large‐scale preparation methods, and cost‐effectiveness remains a major challenge for large‐scale production and practical industrialization of AZMBs. To further rationalize the design of high‐performance ZMAs and advance the practical applications of AZMBs, the perspectives and suggestions for advanced host designs are summarized in Figure 14b and listed as follows.

**TABLE 1:** Comparative summary of CE and performances of symmetric cells using different hosts based on surface modification strategies.

Host	Strategies	CE (%)	Current density (mA cm^−2^)	Plating/stripping capacity (mAh cm^−2^)	Life (h)	Voltage hysteresis (mV)	Ref.
Ag NPs@CC	Surface modification	99.5	2	2	800	56	[150]
ZIF‐8 derived carbon		98.6	1	1	/	/	[160]
Ti_3_C_2_T*_x_* MXene		∼100	1	1	200	22	[67]
Cu NBs@NCFs		98.8	2	1	450	34.6	[162]
Graphene		99.9	40	/	/	/	[153]
Zn/Sn_(200)_		98.8	0.5	1	500	8	[190]
Ti_3_C_2_T*_x_*		94.13	1	1	400	83	[191]
Carbon felt @Zn‐Sn alloy		/	5	5	240	75	[76]
CnC HS		95	4	1	116	20	[151]

**TABLE 2:** Comparative summary of CE and performances of symmetric cells using different hosts based on interface engineering.

Host	Strategies	CE (%)	Current density (mA cm^−2^)	Plating/stripping capacity (mAh cm^−2^)	Life (h)	Voltage hysteresis (mV)	Ref.
ZnO/MG aerogel	Interface engineering	99.2	1	1	500	21.5	[45]
3DP‐NC		99	1	1	380	5.6	[74]
ZnGaIn//MXene		/	1	1	600	25	[161]
3D Ni lattices		/	5	2	200	/	[75]
Zn_88_Al_12_ alloys		∼100	0.5	/	2000	/	[8]
C‐CNT		99.1	1	1	6500	43	[95]
Cu_5_Zn_8_@NC		99.7	1	1	7000	16.5	[50]
HMCOF‐Cu		/	10	1	2500	24.3	[103]

**TABLE 3:** Comparative summary of CE and performances of symmetric cells using different hosts based on spatial confinement strategies.

Host	Strategies	CE (%)	Current density (mA cm^−2^)	Plating/stripping apacity (mAh cm^−2^)	Life (h)	Voltage hysteresis (mV)	Ref.
Sn@NHCF	Spatial confinement	99.7	1	1	370	21	[84]
3D‐ZGC			5	1	1200	35	[80]
3D porous Ti@TiO_2_		95.01	1	1	2000	28	[68]
3D hollow SiO_2_/TiO_2_/carbon fiber		99.54	10	1	600	58	[77]
SDF		/	2	1	1000	20	[184]
N‐VG@CC		95	0.5	0.5	150	6.2	[105]
Cu@CuO nanowires		/	1	1	340	20	[181]
TZNC		98.6	1	1	450	12	[78]

- Rationally screening suitable host materials: The primary criterion for commercially viable hosts lies in excellent electrochemical performance, a large‑scale manufacturable route, and low cost. Lightweight carbon‐based conductive hosts possess prominent merits including high electronic conductivity, robust chemical inertness, abundant natural reserves, and good flexibility. For example, Jin et al. [187] adopted low‐cost graphite paper as a conductive host to realize the large‐scale preparation of high‐performance ZMAs. The cost‐effective graphite‐paper‐based ZMAs enable the assembled Zn||I_2_ multilayer pouch battery with an Ah‐level capacity and long‐term cycling stability. Benefiting from mature industrial manufacturing routes, robust chemical inertness and low cost, metallic Cu has been widely adopted as a conductive host for lithium‐ion and Zn‐ion batteries. Wu et al. [188] realized the low‐cost, scalable fabrication of nanotwinned copper (ntCu) via a facile electrodeposition strategy. Featuring outstanding electrical conductivity and exceptional mechanical robustness, the ntCu scaffold endows Zn@P‐ntCu electrodes with superior dendrite‐free cycling performance and long‐term durability. Conversely, Al‐ or Ni‐based hosts exhibit strong zincophilicity, while they suffer from severe corrosive degradation even in weakly acidic electrolytes owing to their high electrochemical activity. Moreover, Ni‐based hosts usually deliver high catalytic activity toward the HER, which triggers severe parasitic side reactions. Furthermore, the excessively high cost of Ti‐based and Ag‐based hosts severely restricts their practical applications. Based on the foregoing research progress, researchers need to integrate advanced host fabrication techniques, including electrospinning, 3D printing, and magnetron sputtering, to further optimize the manufacturing procedures, so as to meet the standards for industrial mass production.
- Theoretical guidance of host design: An unambiguous theoretical framework is indispensable for guiding the optimization of host scaffold design and scalable industrial fabrication routes. The fundamental understanding of the mechanism of the electrode process of ZMAs is still in its infancy. For example, the theoretical models and simulations built for studying the ZMAs electrode process are mostly based on Zn electrodeposition at present, which can not comprehensively explain the fundamental principle of the whole deposition/dissolution process. Apart from Zn electrodeposition, the fundamental principles of Zn dissolution are equally important and still insufficient. Hence, Zn dissolution behavior should be considered under different conditions. Therefore, it is necessary to carry out an in‐depth investigation of the electrode process in terms of thermodynamics and kinetics. In view of the above, employing in situ characterization techniques to systematically investigate the morphological and chemical evolution of ZMAs within the 3D host during the Zn deposition process is crucial. Theoretical simulations can be further introduced to fundamentally unravel the mechanisms of host architecture and compositions on Zn plating behavior, enabling the rational optimization of host design. In addition, machine learning and artificial intelligence approaches can facilitate the high‐throughput screening of host materials, structures, and electrochemical performances, so as to design and fabricate ZMAs that satisfy the commercialization requirements of AZMBs.
- The commercialization of AZMBs: The exploitation and utilization of composite ZMAs using 3D conductive hosts are still at an initial stage in the laboratory, limited by the complexity of the synthesis process, which hampers the commercial application to some extent. Moreover, the assessment systems of battery performance in the laboratory differ substantially from the industrial standards conventionally used in batteries. Firstly, excess metallic Zn leads to an excessively high negative‐to‐positive (N/P) capacity ratio and inferior Zn utilization. As a consequence, AZMBs deliver a practical energy density drastically inferior to the theoretical value. Developing rational design strategies for 3D conductive hosts to boost the utilization efficiency of ZMAs or achieve anode‐free aqueous AZMBs is critical for reducing the N/P ratio, elevating the battery energy density, and accelerating the commercialization of AZMBs. Moreover, large‐scale industrialization of aqueous AZMBs demands the exploitation of synergistic protection strategies for ZMAs. Synergistic integration of electrolyte optimization strategies (e.g., high‐concentration electrolyte and functional additives) enables effective mitigation of parasitic reactions, which substantially boosts both the CE and long‐term cycling stability of ZMAs.

## Author Contributions

**Peng Xiao Sun**: conceptualization, investigation, Writing – original draft. **Quanwei Ma**: conceptualization, writing – original draft. **Sen Xin**: supervision, writing – review and editing. **Yangyang Liu**: writing – original draft, investigation. **Le Yu**: supervision, writing – review and editing. **Yu‐Hui Zhu**: investigation. **Rui Wang**: investigation. **Longhai Zhang**: writing – original draft, investigation. **Chaofeng Zhang**: supervision, writing – review and editing, resources. **Tengfei Zhou**: supervision, writing – review and editing.

## Funding

Natural Science Foundation of Beijing Municipality (Grant No. L223008), Joint funding of BUCT‐CNPC New Energy and New Materials Innovation Consortium (Grant No. PRIKY23106), National Key R&D Project (Grant No. 2024YFE0101100) under its Singapore‐China Joint Flagship Project (Clean Energy), and National Natural Science Foundation of China (Grant Nos. 92472120, 22421001, 52172252, and 22278019). Anhui Postdoctoral Scientific Research Program Foundation (2025A1029), Postdoctoral Fellowship Program of CPSF under Grant (GZC20250098), China Postdoctoral Science Foundation‐Anhui Joint Support Program (2024T013AH).

## Conflicts of Interest

The authors declare no conflict of interest.

## Data Availability

Data sharing not applicable to this article as no datasets were generated or analyzed during the current study.
